# Development of a PET radioligand for potassium channels to image CNS demyelination

**DOI:** 10.1038/s41598-017-18747-3

**Published:** 2018-01-12

**Authors:** Pedro Brugarolas, Jorge E. Sánchez-Rodríguez, Hsiu-Ming Tsai, Falguni Basuli, Shih-Hsun Cheng, Xiang Zhang, Andrew V. Caprariello, Jerome J. Lacroix, Richard Freifelder, Dhanabalan Murali, Onofre DeJesus, Robert H. Miller, Rolf E. Swenson, Chin-Tu Chen, Peter Herscovitch, Daniel S. Reich, Francisco Bezanilla, Brian Popko

**Affiliations:** 1 Department of Neurology, University of Chicago, Chicago, IL USA; 2 Department of Biochemistry and Molecular Biology, University of Chicago, Chicago, IL USA; 3 Department of Radiology, University of Chicago, Chicago, IL USA; 4 Imaging Probe Development Center, NIH/NHLBI, Bethesda, MD USA; 5 Department of Neurosciences, Case Western Reserve University, Cleveland, OH USA; 6 Department of Medical Physics, University of Wisconsin at Madison, Madison, WI USA; 7 Positron Emission Tomography Department, NIH/CC, Bethesda, MD USA; 8 Translational Neuroradiology Section, NIH/NINDS, Bethesda, MD USA; 9 Present Address: Massachusetts General Hospital, Boston, MA USA; 10 Present Address: Universidad de Guadalajara, Guadalajara, Jalisco Mexico; 11 Present Address: University of Calgary, Calgary, Alberta Canada; 12 Present Address: Western University of Health Sciences, Pomona, CA USA; 13 Present Address: George Washington University, Washington, DC USA

## Abstract

Central nervous system (CNS) demyelination represents the pathological hallmark of multiple sclerosis (MS) and contributes to other neurological conditions. Quantitative and specific imaging of demyelination would thus provide critical clinical insight. Here, we investigated the possibility of targeting axonal potassium channels to image demyelination by positron emission tomography (PET). These channels, which normally reside beneath the myelin sheath, become exposed upon demyelination and are the target of the MS drug, 4-aminopyridine (4-AP). We demonstrate using autoradiography that 4-AP has higher binding in non-myelinated and demyelinated *versus* well-myelinated CNS regions, and describe a fluorine-containing derivative, 3-F-4-AP, that has similar pharmacological properties and can be labeled with ^18^F for PET imaging. Additionally, we demonstrate that [^18^F]3-F-4-AP can be used to detect demyelination in rodents by PET. Further evaluation in Rhesus macaques shows higher binding in non-myelinated *versus* myelinated areas and excellent properties for brain imaging. Together, these data indicate that [^18^F]3-F-4-AP may be a valuable PET tracer for detecting CNS demyelination noninvasively.

## Introduction

Myelin is the multilayered lipid membrane that wraps axons and serves to enhance neuronal conduction velocity. Integrity of the myelin sheath is critical for proper function of the nervous system and its disruption has been implicated in a myriad of CNS disorders including multiple sclerosis (MS)^1^, leukodystrophies^2^, traumatic brain and spinal cord injuries^3,4^, progressive multifocal leukoencephalopathy (PML)^5^, and even maladies not traditionally associated with demyelination such as brain ischemia^6^, psychiatric disorders^7^, and neurodegenerative diseases^8^. Nevertheless, the extent to which myelin abnormalities contribute to CNS dysfunction has been difficult to assess due to the lack of direct, noninvasive imaging approaches to quantify changes in myelin.

Currently, myelin disorders are primarily imaged using magnetic resonance imaging (MRI). MRI, which relies on measuring changes in spin-relaxation times of water protons, offers excellent spatial resolution and good contrast in soft tissues but lacks specificity to distinguish and quantify specific biochemical processes such as demyelination, inflammation, and axonal loss^9^. Unlike MRI, positron emission tomography (PET), which is based on the detection of a radioactive drug, is quantitative and offers excellent biochemical specificity, as well as exceptional sensitivity. Therefore, PET can provide complementary information to MRI by revealing the specific underlying biochemical processes involved. For some of these processes, such as neuroinflammation, which is often present but not unique to demyelinating diseases, there are already multiple well-validated tracers, such as ligands for the 18 kDa translocator protein (TSPO)^10–16^. Nevertheless, it remains challenging to image demyelination by PET.

The myelin sheath is composed of lipids (70% of dry weight) and proteins. All existing PET tracers in use to evaluate demyelination consist of hydrophobic molecules proposed to bind to myelin proteins^17–22^. Consequently, these radioligands display a high baseline signal in normal white matter (myelin-rich areas), making it difficult to detect small areas of demyelination. We posited that a PET tracer that specifically binds to a protein on demyelinated axons, thus providing a positive readout for demyelination, would have major advantages over current tracers. Upon demyelination, axonal potassium (K^+^) channels, which normally reside at the juxtaparanodal region beneath the myelin sheath (*e.g*., K_v_1.1 and K_v_1.2), become exposed, disperse throughout the internodes, and increase in expression^23–26^. This aberrant distribution of K^+^ channels causes leakage of intracellular K^+^ ions and renders neurons unable to fully depolarize and propagate action potentials. The clinically approved drug for MS, 4-aminopyridine (4-AP), binds to and blocks these channels, reducing the aberrant efflux of K^+^ ions and restoring conduction of demyelinated axons^27–31^.

Thus, we decided to investigate whether a radiolabeled derivative of 4-AP could serve as a PET tracer for imaging changes in myelination. In this study, we first examined the distribution of 4-AP in the brain of control and demyelinated mice; we then synthesized and screened several fluorinated derivatives of 4-AP compatible with PET imaging; and, finally, we labeled the most promising compound with ^18^F and conducted PET imaging in rodents and non-human primates.

## Results

### Distribution of 4-AP in WT and dysmyelinated mice

Although a number of electrophysiological studies have shown that 4-AP exerts its benefit by binding to voltage-gated K^+^ channels in demyelinated axons^27,32^, its distribution at the macroscopic level is unknown. To determine the potential utility of 4-AP as a tracer, we evaluated its distribution in the brains of control and dysmyelinated mice after *in vivo* administration. To do this, ^14^C-labeled 4-AP was injected intravenously into unanesthetized wild type mice and mice lacking compact myelin due to a null mutation in the gene encoding myelin basic protein (Shiverer, *Mbp*^*shi/shi*^)^33–38^. Thirty minutes post-injection of the radiolabeled drug, the mice were euthanized and their brains dissected without perfusion to prevent washout of the drug. After dissection, the brains were frozen, cut into thin sections and collected on glass slides. The brain slices were then apposed to autoradiography film to reveal the distribution of the drug in the brain at the time of death. After autoradiography, the same brain sections were stained with the myelin-specific dye Luxol fast blue (LFB).

**Figure 1:**
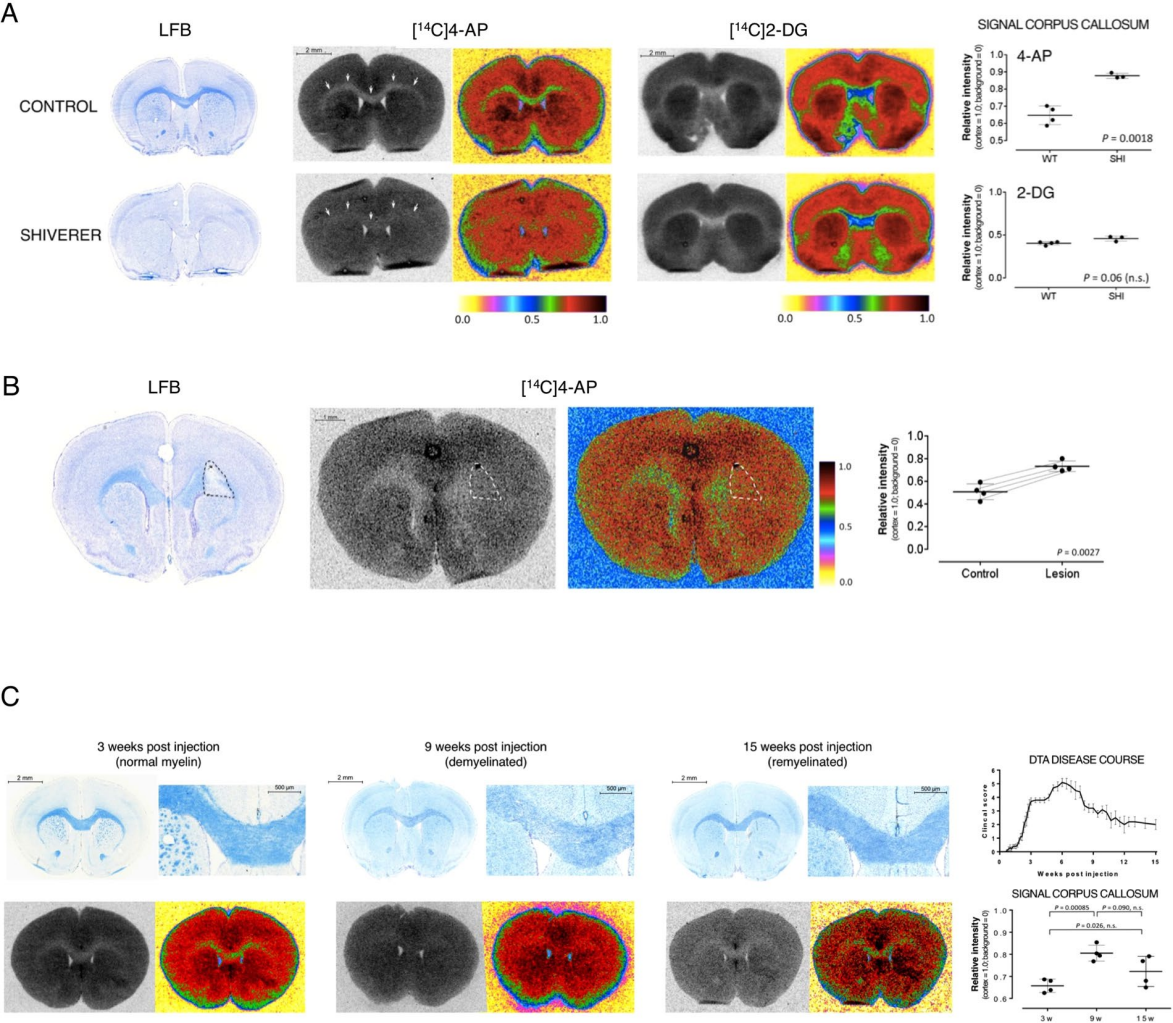
Increased binding of [^14^C]4-AP in white matter areas of dysmyelinated and demyelinated mouse brains. (**A**) Increased binding of 4-AP in Shiverer mice. *Left:* LFB staining showing lack of myelin in the Shiverer mouse. *Middle:* [^14^C]4-AP autoradiography. The same sections were used for autoradiography and LFB staining. The corpus callosum is marked by arrows. False color images are shown to highlight the differences in uptake. Right: [^14^C]2-DG autoradiography and false color image. *Dot plot*: quantification of the autoradiographic signal intensity in the corpus callosum. The cortex was used to normalize across animals. Line represents mean value and error bars represent standard deviation. *P* value was calculated using two-tail Student’s *t*-test (α = 0.05, n.s. = non significant). (**B**) Increased binding of [^14^C]4-AP in focal lesions produced by injection of lysolecithin. *Left:* LFB staining showing focal demyelination on the right side of the corpus callosum (dashed line). A black charcoal spot left by the syringe shows the site of injection. *Middle:* [^14^C]4-AP autoradiography and false color image. *Dot plot*: quantification of the autoradiographic signal intensity in the lesion and contralateral control areas. Each pair of dots connected by a thin line represents one animal, ≥6 sections quantified per animal. The cortex was used to normalize across animals. Horizontal line represents mean value and error bars represent 95% confidence interval. P value calculated using two-tail Student’s *t*-test (α = 0.05, paired data). (**C**) Changes in binding of [^14^C]4-AP in DTA mice. DTA mice show prominent demyelination 9 weeks post injection of tamoxifen and robust remyelination 15 weeks post injection. *Left:* LFB staining showing normal myelin at 3 weeks post injection, severe demyelination 9 weeks post injection and robust significant remyelination 15 weeks post injection. *Middle:* [^14^C]4-AP autoradiography and false color image. *Dot plot*: quantification of the autoradiographic signal intensity in the corpus callosum. The cortex was used to normalize across animals. Each dot represents one animal, ≥6 sections quantified per animal. Line represents mean value and error bars represent 95% confidence interval. P value calculated using ANOVA (α = 0.05). *Right*: scatter plot showing disease course (clinical score as follows: 0 = no symptoms, 1 = flaccid tail, 2 = mild ataxia, 3 = severe ataxia and splayed gait, 4 = severe ataxia plus tremor plus dragging of hind limbs, 5 = severe ataxia plus tremor plus problems righting themselves, and 6 = immobile, laterally recumbent).

In wild-type mice, areas of high concentration of myelinated axons (white matter) such as the corpus callosum and anterior commissure appear intensely blue when stained with LFB (Fig. 1A). [^14^C]4-AP was present at low levels in these white matter areas (relative intensity = 0.647 ± 0.046, n = 4) compared to non-myelinated gray matter areas (relative intensity = 1.0, n = 4). In contrast, Shiverer mice showed high radioactive signal in dysmyelinated white matter areas such as the corpus callosum (relative intensity = 0.878 ± 0.013, n = 3, *P* = 0.0018). This represents an increase in signal of 33%. It is important to note that this regional distribution is observed only when [^14^C]4-AP is administered *in vivo*. If the drug is applied directly onto brain sections *in vitro*, low signal is seen uniformly across the whole tissue. This finding is consistent with the established mechanism that requires a membrane potential for the K^+^ channels to open and the drug to bind.

The distribution pattern of 4-AP found in wild type mice is similar to that of kaliotoxin, a selective blocker of K_v_1.1, K_v_1.2 and K_v_1.3 channels^39^, consistent with the hypothesis that 4-AP targets K^+^ channels. Nevertheless, this distribution pattern is also similar to that of 2-deoxy-*d*-glucose, 2-DG, and its analog, the commonly used PET tracer [^18^F]2-fluoro-2-deoxy-*d*-glucose ([^18^F]FDG). The uptake of 2-DG in the brain correlates with tissue metabolism and is not expected to correlate with changes in myelin. To ensure that the differences seen with 4-AP were specific to changes in myelin and not due to changes in brain metabolism in the Shiverer mouse, we evaluated the distribution of [^14^C]2-DG using the same autoradiographic technique (Fig. 1A). This experiment produced the expected distribution of 2-DG in control brains: high binding in gray matter and low binding in white matter^40^, confirming the validity of the autoradiographic method employed. Importantly, no differences were seen in the distribution of 2-DG between wild type and Shiverer mice, confirming that the differential binding of 4-AP in the brains is not due to changes in metabolism, but to changes in myelin.

### Distribution of 4-AP in focally demyelinated mouse brains

Focal demyelinated lesions are commonly seen in MS patients. To extend our results to a model that would better capture this feature, we used the lysolecithin (LPC) injection model. In this model, intracranial injection of LPC results in focal demyelination at the site of injection^41,42^. Injection of LPC into the corpus callosum of one hemisphere provides a system in which one side of the corpus callosum is demyelinated while the contralateral uninjected side remains normally myelinated^43^. Three days after injection of LPC, [^14^C]4-AP was administered and the distribution of the radiolabeled drug in the brain evaluated using *ex vivo* autoradiography. The autoradiographic signal derived from [^14^C]4-AP in the demyelinated area was compared to a similar area in the opposite hemisphere. As in the previous model, the signal in the demyelinated area was significantly higher than in the control area (0.733 ± 0.047 *vs*. 0.507 ± 0.070, n = 4, *P* = 0.0027) (Fig. 1B). Once the autoradiography of these brain sections was completed, we stained the same sections with LFB and found that the regions of the corpus callosum with more intense autoradiographic signal clearly correlated with demyelinated areas as identified by the absence of LFB staining.

### 4-AP in a mouse model of demyelination and remyelination

The CNS has a robust potential to remyelinate following demyelinating insults^44^. To assess the potential of 4-AP to distinguish between demyelinated and remyelinated lesions we used the DTA model of oligodendrocyte ablation^45^. The DTA model is a genetically engineered mouse strain in which injection of tamoxifen indirectly induces expression of diphtheria toxin A in oligodendrocytes, resulting in cell death and widespread CNS demyelination. In this model, axons are spared, and demyelination is followed by robust remyelination.

For this experiment, we evaluated the uptake of 4-AP at three different time points: prior to demyelination (3 weeks post tamoxifen injection), during severe demyelination (9 weeks post tamoxifen), and during remyelination (15 weeks post tamoxifen). These time points were chosen based on previous reports on this model^45,46^, as well as histological analysis and evaluation of clinical symptoms (Fig. 1C). As seen in figure 1C, at three weeks post tamoxifen there was low uptake of [^14^C]4-AP in the corpus callosum (relative signal = 0.657 ± 0.031, n = 4) consistent with normal myelination. At nine weeks post injection, there was an increase in the uptake of [^14^C]4-AP in the corpus callosum (relative signal = 0.805 ± 0.036, n = 4, *P* = 0.00085) consistent with the demyelination observed on the LFB staining. Fifteen weeks post tamoxifen, the level of [^14^C]4-AP in the corpus callosum was in between peak demyelination and baseline (relative intensity = 0.739 ± 0.070, n = 4) which is consistent with the partial remyelination observed by LFB staining. This experiment showed that the uptake of 4-AP has potential for assessing changes due to demyelination and remyelination.

As demyelination is also common in the spinal cord, we evaluated the distribution of 4-AP in the spinal cord of one control and one DTA mice. In the control, we saw clear lines of low signal running throughout the length of the spinal cord, which correspond to the dorsal and ventral white matter columns. In the DTA, there was high signal in these areas, which correlated with demyelinated areas on LFB staining (Sup. Fig. 1).

### Choice of PET ligand: fluorinated derivatives of 4-AP

Having shown that 4-AP preferentially localizes to demyelinated and non-myelinated areas in the mouse brain, our next step in developing a PET tracer involved creating a derivative of 4-AP amenable to labeling with a positron-emitting radionuclide. The most common positron-emitting radionuclides for small molecule tracers are carbon-11 and fluorine-18. We chose to pursue ^18^F-labeled derivatives because fluorine-18 has longer half-life than carbon-11 (109 min *vs*. 20.4 min), which greatly facilitates its use.

**Figure 2:**
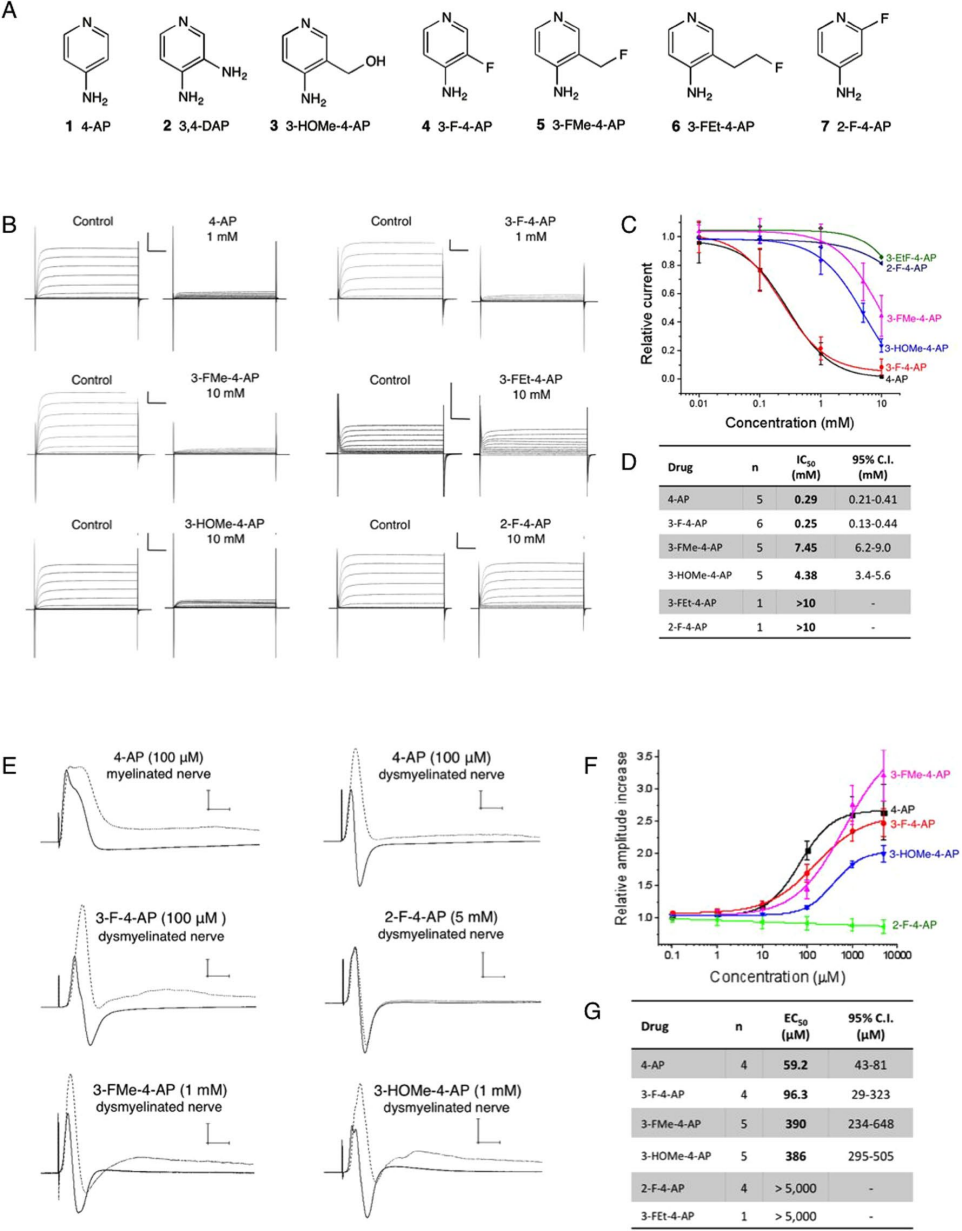
Fluorinated derivatives of 4-AP. (**A**) Compounds tested in this study: (1) 4-aminopyridine. (2) 3,4-diaminopyridine. (3) 3-hydroxymethyl-4-aminopyridine. (4) 3-fluoro-4-aminopyridine. (5) 3-fluoromethyl-4-aminopyridine. (6) 3-fluoroethyl-4-aminopyridine. (7) 2-fluoro-4-aminopyridine. (**B**) Drug binding to K^+^ channels expressed in frog oocytes: K^+^ currents were generated by a series of 50 ms pulses from −70 mV to +40 mV in increments of 10 mV in the presence of cumulative concentrations of 4-AP derivatives. Each panel represents the K^+^ current recorded from the same oocyte before and after addition of each drug. Scale bar: 1 μA/10 ms. (**C**) Relative K^+^ current *vs*. concentration for each drug obtained at +20 mV. (**D**) Half-maximal inhibitory concentration of each molecule and 95% confidence interval obtained from fitting the data to the Hill equation. n = number of times each drug was tested in separate oocytes. (**E**) Drug binding to K^+^ channels in explanted mouse optic nerves: compound action potential (CAP) traces before (solid line) and after (dashed line) addition of each drug. Scale bar: 5 mV/5 ms. (**F**) Relative increase of maximum CAP amplitude *vs*. concentration for each drug. Amplitude was normalized to the amplitude before the drug. (**G**) Half-maximal effective concentration of each molecule and 95% confidence interval obtained from fitting the data to the Hill equation. n = number of times each drug was tested in separate nerves.

Several 4-AP related structures are shown in figure 2A. From these, 3,4-diaminopyridine (3,4-DAP, **2**) and 4-amino-3-hydroxymethylpyridine (3-HOMe-4-AP, **3**) are known to retain the capacity to bind to K^+^ channels^47–50^. Thus, we decided to investigate the following fluorinated derivatives: 2-fluoro-, 3-fluoro-, 3-fluoromethyl- and 3-fluoroethyl-4-aminopyridine (**4**–**7**). We also included 4-AP and 3-OHMe-4-AP (**1** and **3**) for comparative purposes. Of these compounds, **1** through **5** are commercially available, while **6** and **7** were synthesized by deoxyfluorination of the respective alcohols as described in the supplementary methods.

### Blockage of K^+^ channels by 4-AP derivatives under voltage clamp conditions

After obtaining the 4-AP derivatives, we tested their ability to block voltage-gated K^+^ channels expressed in *Xenopus* oocytes using the cut-open voltage clamp technique described by Stefani and Bezanilla^51^. For this experiment, we selected the non-inactivating Shaker channel from *D. melanogaster*, as it is the most commonly studied voltage-gated K^+^ channel^52,53^. This channel shares an identity ranging from 69–79% with neuronal K_v_1.1, K_v_1.2, K_v_1.4 and K_v_1.6 that are among the presumed targets of 4-AP and has comparable sensitivity to 4-AP^54,55^. In order to assess the relative potency of the different 4-AP derivatives, each drug was applied at increasing concentrations, and the ratio between the K^+^ current with and without drug was computed (Fig. 2B,C). 4-AP and 3-F-4-AP displayed the most potent effects with half-maximal inhibitory concentrations (IC_50_) around 0.27 mM (4-AP: IC_50_ = 0.29 mM, 95% confidence interval (CI_95_) = 0.21–0.41 mM; 3-F-4-AP: IC_50_ = 0.25 mM, 95.0% C.I. = 0.13–0.44 mM). 3-HOMe-4-AP and 3-FMe-4-AP were between 15 and 25 times less potent than 4-AP (3-HOMe-4-AP: IC_50_ = 4.38 mM, CI_95_ = 3.4–5.6 mM; 3-FMe-4-AP: IC_50_ = 7.45 mM, CI_95_ = 6.2–9.0 mM). In contrast, 3-FEt-4-AP and 2-F-4-AP had IC_50_ values greater than 10 mM (CI_95_ not determined). These results demonstrate that small modifications in the 3 position of 4-AP are permitted, whereas large modifications and substitutions in the 2 position significantly diminish potency. We also observed that the effect of these drugs on the ionic current persisted after several minutes of continuous flow of buffer that did not contain any drug. This effect has previously been reported for 4-AP^55^ and indicates that drug gets trapped inside the protein when the channel closes. Since the drug becomes trapped inside the channel, the dissociation constant (*k*_off_) is predicted to be very slow and therefore the binding constant (*K*_d_ = *k*_on_/k_off_) very high. Thus, the IC_50_ value measured above does not represent the *K*_d_ but rather an experimental value useful for comparing the relative potency of the different derivatives. In fact, the concentration of 4-AP in the cerebrospinal fluid of patients taking the drug is estimated around 50 nM^56,57^, suggesting a high binding affinity *in vivo*.

### Effects of 4-AP derivatives on the compound action potential of dissected optic nerves

The previous experiment demonstrated that in a simple experimental system with only one type of K^+^ channel, 3-F-4-AP is pharmacologically the most similar analog of 4-AP. We next wanted to verify that the fluorinated 4-AP derivatives retain the capacity to bind to K^+^ channels in a more complex and biologically relevant experimental system^27,58^. Therefore, we tested the effects of these compounds on the compound action potential (CAP) of dysmyelinated optic nerves from control and Shiverer mice (Fig. 2E). In the absence of drug, this experiment showed the typical differences between normally myelinated nerves and dysmyelinated nerves. The Shiverer nerves conducted slower (average conduction velocity: 0.59 ± 0.10 m/s (n = 7) *vs*. 1.40 ± 0.30 m/s (n = 11) at 22.2 ± 1.3 °C, *P* < 0.00001), had smaller amplitude (20–30% compared to myelinated nerves) and showed a larger hyperpolarization than control nerves. Addition of 4-AP to normally myelinated nerves caused small increases in amplitude and duration of the action potential (<5%), suggesting, as previously reported, that K^+^ channels in myelinated fibers are mostly inaccessible to 4-AP^32^. In comparison, addition of 4-AP to dysmyelinated nerves caused significant increases of the CAP amplitude (2–4 fold), generating a CAP that, although delayed, appeared similar to control myelinated nerves. These effects are characteristic of binding to voltage-gated K^+^ channels^58^. In this experiment, 4-AP and 3-F-4-AP were found to be the most potent in enhancing the CAP with half-maximal effective concentrations (EC_50_) of 59.2 μM (CI_95_ = 43–81 μM) and 96 μM (CI_95_ = 29–323 μM), respectively (Fig. 2F,G). The derivatives 3-HOMe-4-AP and 3-FMe-4-AP were about 4–6 times less potent with EC_50_’s about 390 μM (3-HOMe-4-AP: IC_50_ = 386 μM, CI_95_ = 295–505 μM; 3-MeF-4-AP: IC_50_ = 386 μM, CI_95_ = 234–648 μM). Notably, 2-F-4-AP and 3-FEt-4-AP were found to be inactive. The trend observed in this experiment is consistent with the trend observed in the voltage-clamp experiment described above. The differences in absolute values are likely due to differences in experimental condition (*e.g*., pH, voltage) as well as differences between the mouse K_v_1 channels and the Shaker channel. Again, the EC_50_ values in this functional assay do not represent the binding affinity *in viv*o.

### Pharmacology and *in vivo* effects of 3-F-4-AP and 4-AP

**Figure 3:**
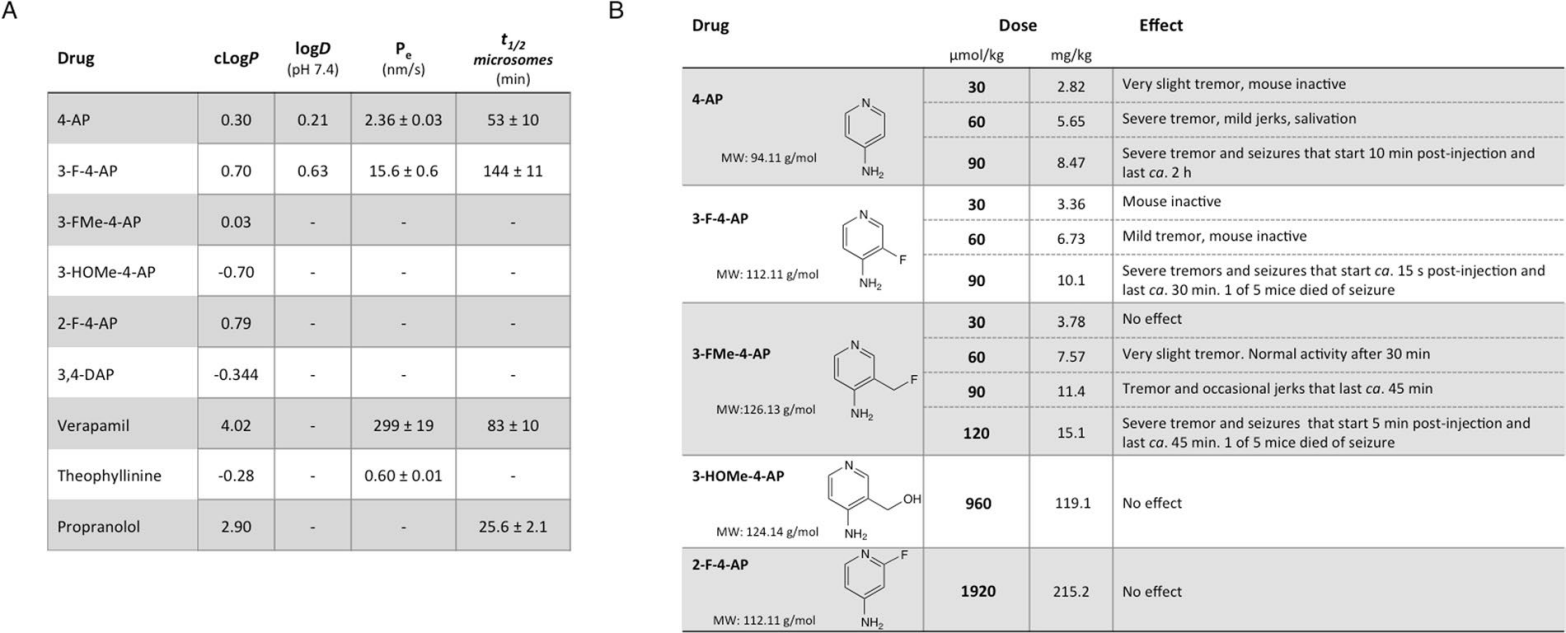
Pharmacology of 4-AP derivatives. (**A**) Pharmacological parameters for 4-AP derivatives and control compounds. cLog*P*: predicted logP value from SciFinder calculated using Advanced Chemistry Development (ACD/Labs) Software V11.02. log*D*: experimental partition coefficient octanol:water at pH 7.4 (n = 3). P_e_: permeability coefficient across artificial membrane (n = 3). *t*_*1/2(microsomes)*_: half-life in mouse microsomes (n = 3). (**B**) *In vivo* effects after intraperitoneal injection of 4-AP derivatives (200 μL/200 g mouse).

Fluorination of drugs can improve stability and brain permeability^59,60^, which are important requirements for developing tracers for the brain. We tested the permeability of 3-F-4-AP and 4-AP to an artificial membrane made of porcine brain lipids (Fig. 3A). In this experiment, highly permeable verapamil and low permeable theophylline were included as controls. We found 3-F-4-AP to be 6.6 times more permeable than 4-AP (P_e_: 15.6 ± 0.6 nm/s *vs*. 2.36 ± 0.03 nm/s, *P* = 0.00034). This value correlates well with the measured partition coefficients in octanol/water for these drugs (log*D* pH 7.4: 0.63 *vs*. 0.23, Pearson r = 0.997, *P* = 0.0033).

We also tested the stability of these drugs in mouse plasma and mouse liver microsomes. Liver microsomes contain large amounts of cytochrome P450 and can be used to gauge the metabolic stability of drugs. In this experiment, highly stable verapamil and minimally stable propranolol were included as controls. Both 4-AP and 3-F-4-AP were found to be stable in plasma (>90% remaining after 1 h) and in liver microsomes (4-AP: *t*_1/2_ (microsomes) = 53 ± 10 min; 3-F-4-AP: *t*_1/2_ (microsomes) = 144 ± 11 min, Fig. 3A). This result indicates that [^18^F]3-F-4-AP is likely to be stable *in vivo*, limiting the number of potentially confounding radiometabolites that may be generated *in vivo*.

At high doses, 4-AP is known to cause tremors, muscle spasms and seizures^61–65^. These effects derive from the excessive blockade of K^+^ channels in the CNS and can be used to assess whether the drugs are active *in vivo*. In this experiment, we injected each drug intraperitoneally at different concentrations into mice and looked for signs of neuroexcitation (Fig. 3B). In agreement with the electrophysiology and permeability experiments, 3-F-4-AP was found to be active at the same concentration as 4-AP and to act faster. 3-FMe-4-AP was found to be half as potent as 4-AP, likely due to the combination of having lower affinity to the channel but greater brain permeability. In contrast, 2-F-4-AP and 3-HOMe-4-AP did not cause any effects at doses up to 20 times greater. The lack of effect of 2-F-4-AP can be explained by the lack of binding to the channel, as demonstrated in the previous experiments, whereas the lack of effect of 3-HOMe-4-AP is likely due to its inability to cross the blood-brain barrier (BBB) as predicted by its cLog*P* of −0.57 (Fig. 3A). It should be noted that at the doses commonly used for PET imaging (<10 μg/subject), the risk of seizures with 3-F-4-AP is negligible. Taken together, these results demonstrate that 3-F-4-AP efficiently penetrates into the CNS and binds to its target *in vivo*, supporting further development as a PET tracer for the brain.

### 3-F-4-AP in the brain of three mouse models of demyelination

**Figure 4:**
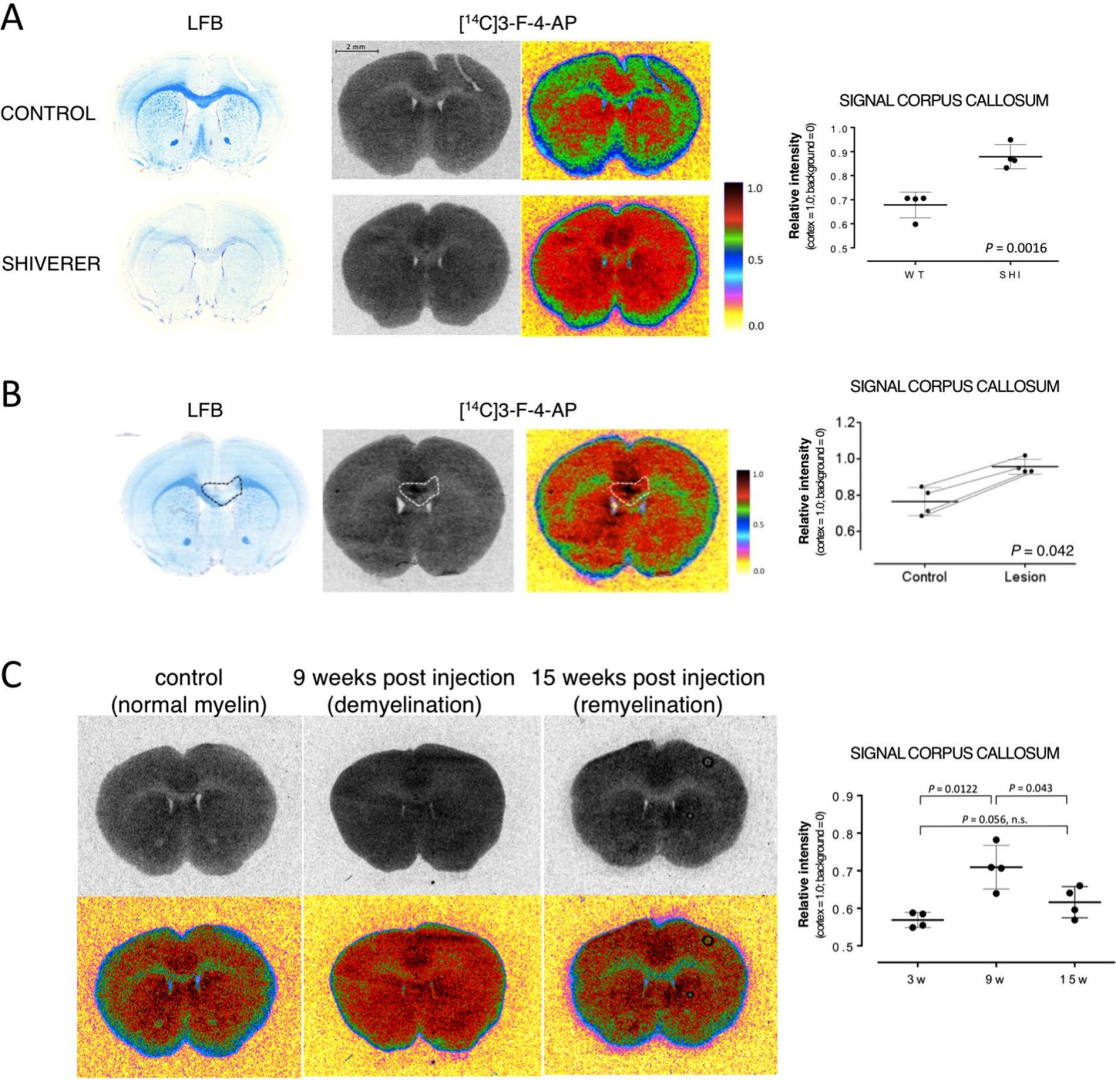
Increased binding of [^14^C]3-F-4-AP in mouse models of MS. (**A**) Increased binding of [^14^C]3-F-4-AP in the corpus callosum of the Shiverer mouse. *Left:* LFB staining showing lack of myelin in the Shiverer mouse. *Middle:* [^14^C]3-F-4-AP autoradiography with false color image to highlight differences. *Dot plot*: quantification of the autoradiographic signal intensity in the corpus callosum. The cortex was used to normalize across animals. Each dot represents one animal, ≥ 6 sections quantified per animal. Line represents the mean and error bars represent 95% confidence interval. *P* value calculated using two-tail Student’s *t*-test (α = 0.05). (**B**) Increased uptake of [^14^C]3-F-4-AP in focal lesions produced by injection of lysolecithin. *Left:* LFB staining showing focal demyelination on the middle to right side of the corpus callosum (dashed line). *Middle:* [^14^C]3-F-4-AP autoradiography and false color image. *Dot plot*: quantification of the autoradiographic signal intensity in the lesion and contralateral control areas. Each pair of dots connected by a thin line represents one animal, ≥6 sections quantified per animal. The cortex was used to normalize across animals. Horizontal line represents mean value and error bars represent 95% confidence interval. P value calculated using two-tail Student’s *t*-test (α = 0.05, paired data). (**C**) Changes in uptake of [^14^C]3-F-4-AP in DTA mice. [^14^C]4-AP autoradiography and false color images corresponding to 0–3 weeks post injection of tamoxifen (normal myelin), 9 weeks post injection (demyelination) and 15 weeks post injection (remyelination). *Dot plot*: quantification of the autoradiographic signal intensity in the corpus callosum. Each dot represents one animal, ≥6 sections quantified per animal. The cortex was used to normalize across animals. Line represents mean value and error bars represent 95% confidence interval. P value calculated using ANOVA (α = 0.05).

Once we identified 3-F-4-AP as our lead compound, we evaluated its distribution in mouse brains using *ex vivo* autoradiography. We injected [^14^C]3-F-4-AP via tail vein into control and Shiverer mice followed by autoradiography and LFB staining (Fig. 4). As previously seen with [^14^C]4-AP, [^14^C]3-F-4-AP was found to have the highest binding in non-myelinated gray matter areas (normalized intensity = 1.0, n = 4) and the lowest binding in myelinated areas (relative signal = 0.679 ± 0.046, n = 4). In addition, as with [^14^C]4-AP, the signal in the corpus callosum of Shiverer mice (*Mbp*^*shi/shi*^) was found to be greater than in control mice (relative signal = 0.878 ± 0.043, n = 4, *P* < 0.0016, Fig. 4A). This represents a 29% increase and shows that binding of 3-F-4-AP can serve to distinguish hypomyelinated from normal white matter.

Additional autoradiographic experiments in LPC injected mice demonstrated that [^14^C]3-F-4-AP can distinguish focal demyelinated lesions (Fig. 4B), as was previously shown with [^14^C]4-AP (Fig. 1B). Furthermore, experiments in DTA mice showed that [^14^C]3-F-4-AP can distinguish demyelinated and remyelinated areas of the CNS (Fig. 4C).

### Preparation of [^18^F]3-F-4-AP

After confirming that 3-F-4-AP has adequate pharmacological properties for a brain tracer and can distinguish myelinated *vs*. non-myelinated and demyelinated areas in the mouse brain, we set out to label 3-F-4-AP with ^18^F for PET imaging experiments. Radiochemical synthesis of [^18^F]3-F-4-AP for rodent imaging experiments was performed using a method that produced the tracer with molar activities > 100 mCi/μmol (3.7 GBq/μmol)^66,67^. For non-human primates the tracer was produced using a different method that produced molar activity > 2,000 mCi/μmol (74 GBq/μmol)^68^.

### Organ distribution of the PET tracer in mice

**Figure 5:**
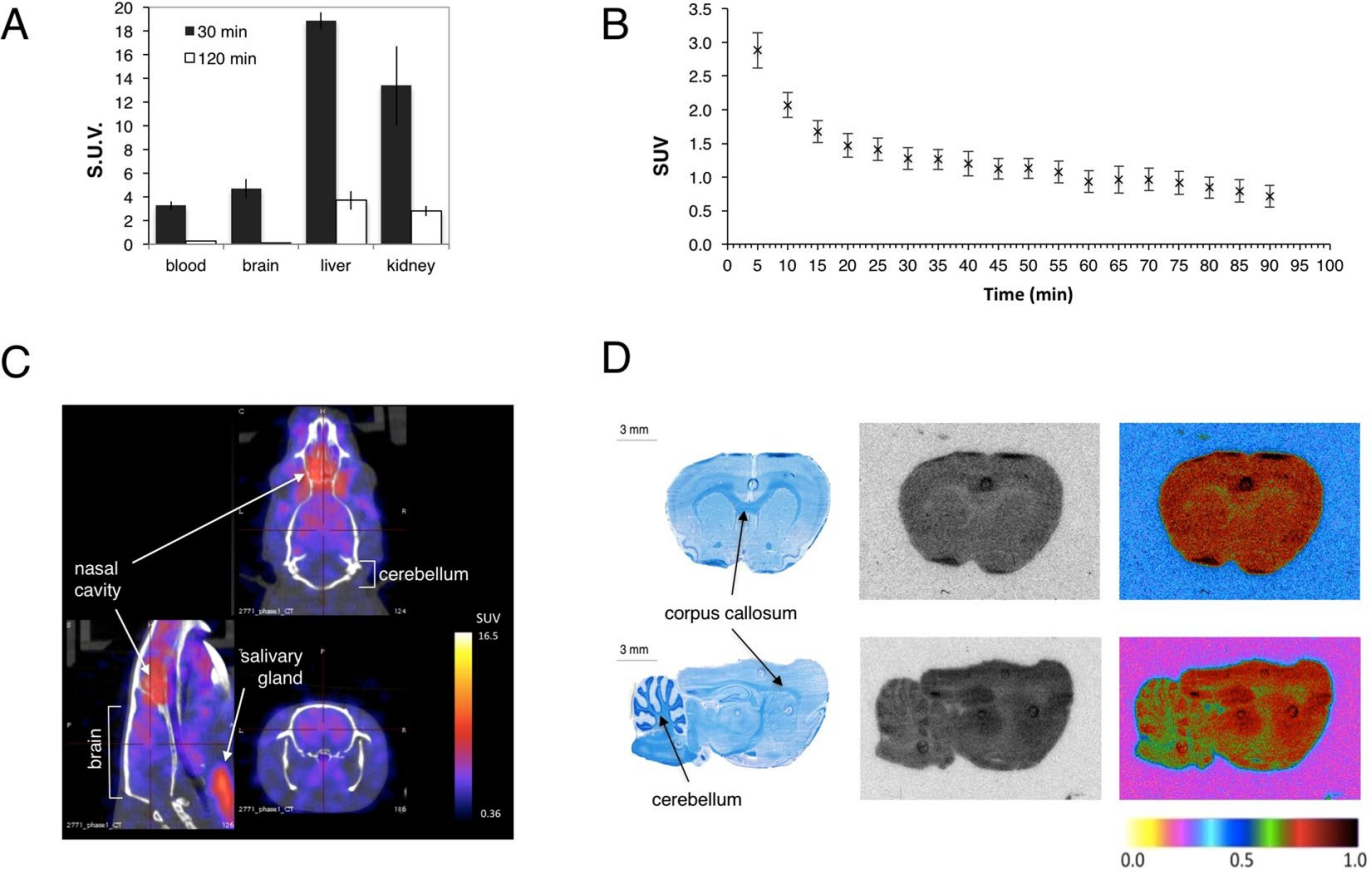
Characterization of [^18^F]3-F-4-AP in control animals. (**A**) Organ distribution in mice measured using gamma counting (n = 4). (**B**) Time activity curves decay-corrected for the time of injection of the rat brain extracted from the quantification of 0–90 min dynamic PET scan in 5 min frames. Sprague Dawley rats received 0.15–0.30 μCi/g of [^18^F]3-F-4-AP via tail vein injection (n = 4, mean ± s.e.m). (**C**) Representative sagittal, axial and coronal fused PET/CT images. PET/CT images of the head were acquired 0 to 30 min post injection. (**D**) Representative LFB and autoradiography images (coronal and sagittal) of rat brains 30 min post injection of [^18^F]3-F-4-AP. Rats received 0.5–1 μCi/g via tail vein injection. There is high correlation between the LFB stain (myelin) and the autoradiography images.

Once we had generated the ^18^F-labeled drug, we evaluated the uptake of the tracer in mouse blood, brain, liver and kidneys 30 min and 2 h post intravenous administration using automatic gamma counting. The results show highest uptake in the kidneys and liver, indicating renal and hepatic clearance of 3-F-4-AP (Fig. 5A). The uptake in the brain 30 min post injection was higher than blood (standard uptake value (SUV) = 4.71 ± 0.81 *vs*. 3.27 ± 0.35, n = 4, *P* = 0.024) providing a suitable time window to conduct PET imaging studies.

### PET imaging, time-activity curves and autoradiography with [^18^F]3-F-4-AP in healthy rats

In order to evaluate the potential use of [^18^F]3-F-4-AP as a PET tracer for the brain, we conducted PET combined with computed tomography (PET/CT) imaging in healthy rats after tail vein injection of [^18^F]3-F-4-AP (0.15–0.30 μCi/g). We chose rats instead of mice because their larger brain size allows better spatial resolution. Time-activity curves extracted from 0 to 90 min scans showed that the tracer has fast entry into the brain (peaks within the first 5 min) followed by a slower washout phase (Fig. 5B). PET/CT images from 0 to 30 min showed radioactivity in the brain with a mean SUV for the whole brain of 2.91 ± 0.65 (n = 3) (Fig. 5C). Although the resolution of the PET images did not allow visualization of small brain areas such as the corpus callosum, it is apparent from the PET quantification that there is higher uptake in non-myelinated areas of the forebrain such as the cortex (SUV = 3.24 ± 0.70) than in myelin-rich areas of the hindbrain such as the cerebellum (SUV = 2.09 ± 0.44), consistent with the proposed mechanism of action. In addition, no radioactivity was detected in the skull, teeth or bone indicating that the tracer does not undergo defluorination *in vivo*, consistent with the microsome stability assay. Attempts to block the signal using high doses of non-radiolabeled 3-F-4-AP (self-block) caused the breathing rate to increase dramatically, possibly due to seizures, and were not pursued further. In order to overcome the limited intrinsic resolution of PET, we conducted *ex vivo* autoradiography in rats injected with [^18^F]3-F-4-AP (1–2 μCi/g). Autoradiographic images of the brain (Fig. 5D) clearly showed a low uptake in white matter areas (*e.g*. corpus callosum, anterior commissure, *arbor vitae* of the cerebellum and brain stem) and high uptake in gray matter areas (*e.g*. cortex, striatum and hippocampus), in agreement with our previous [^14^C]3-F-4-AP autoradiography findings.

### Detection of demyelination by PET using the [^18^F]3-F-4-AP tracer

**Figure 6:**
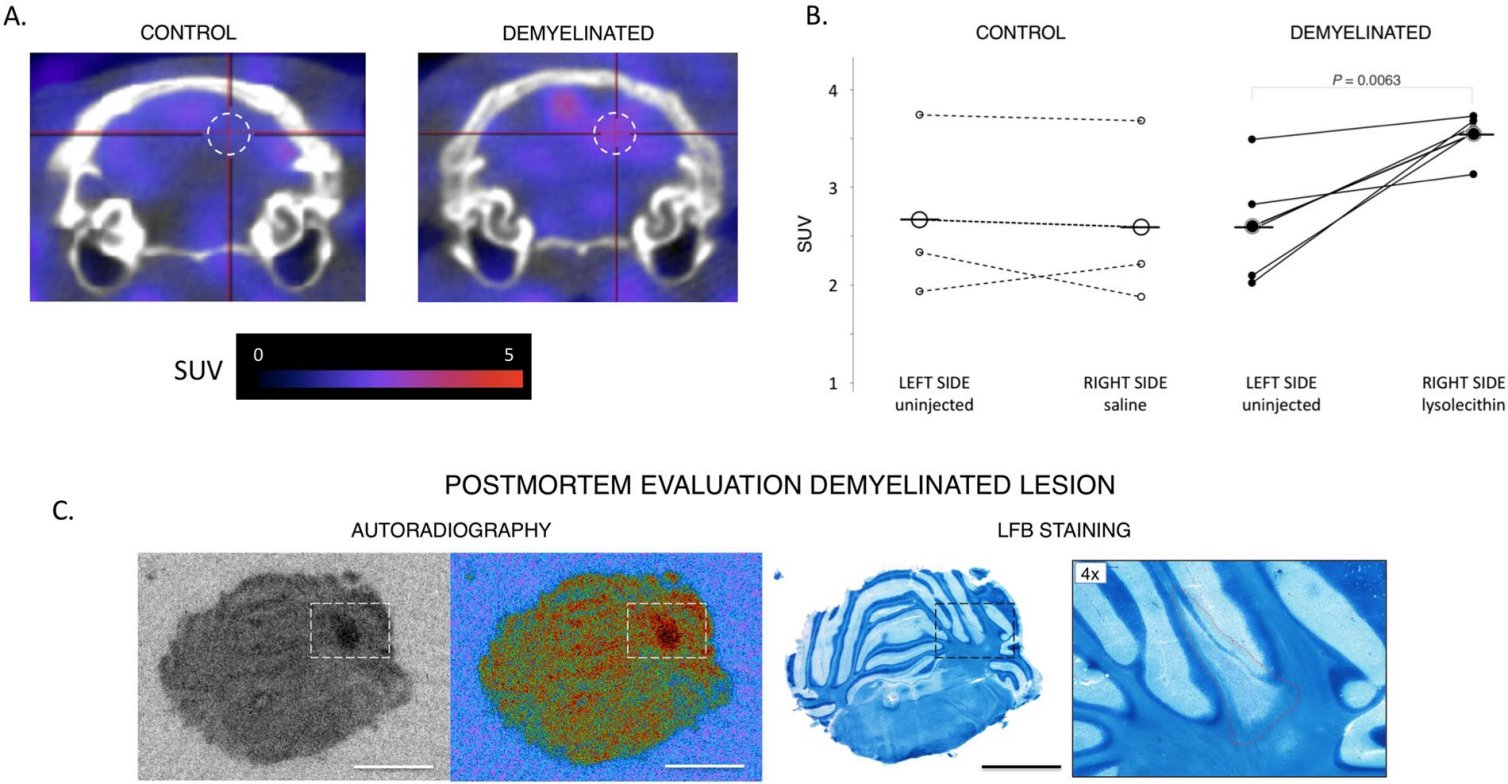
Detection of demyelination by PET and confirmation by autoradiography and histochemical staining. (**A**) Representative coronal fused PET/CT images of the cerebellum of injected rats. Sprague Dawley rats received 0.15–0.30 μCi/g of [^18^F]3-F-4-AP via tail vein injection. PET/CT images were acquired 0 to 25 min post injection. Dashed circle represents region of interest. (**B**) Quantification of the SUV of the lesioned and control regions of interest. There is significant increase in SUV on the on the LPC-injected animals (*P* = 0.0063) but not on the saline-injected animals. (**C**) Representative *ex vivo* autoradiography and LFB staining of LPC injected rat. (Scale bar: 4 mm). There is high correlation between PET, autoradiography and LFB staining.

After conducting PET imaging with [^18^F]3-F-4-AP in healthy rats, we sought to evaluate the capability of this tracer to detect demyelination using the LPC-injection model. The main advantages of this model are that the localization of the lesion is known, that the time course of demyelination is well established, that there is no inflammation or disruption of the BBB, and that the signal in the lesioned area can be directly compared to the contralateral control area^69^. We chose to make the injections in the cerebellum because this myelin-rich region displays low baseline binding of [^18^F]3-F-4-AP. Rats were injected with 3 μL of saline (control group) or 1% LPC in saline (demyelination group) on the right side of the cerebellum, targeting the cerebellar white matter. Seven days post injection, which corresponds to the peak of demyelination, the animals were scanned using PET (0–25 min). Several hours after the PET scan, the animals were reinjected with [^18^F]3F4AP and their brains studied by *ex vivo* autoradiography as previously described. Finally, following autoradiography the same tissue sections were stained with LFB. To analyze the data, we placed a spherical voxel of ~1.5 mm radius centered at the injection coordinates and compared the signal in this region with that of an equal size region at the contralateral hemisphere (Fig. 6A). This experiment shows an increase in signal in the animals that received LPC injections (control side = 2.60 ± 0.21, injected side = 3.54 ± 0.53, n = 4, *P* = 0.010) but not in those that received vehicle only (control side = 2.67 ± 0.77, injected side = 2.59 ± 0.78, n = 3, *P* = 0.38) (Fig. 6B). Furthermore, there was excellent correlation between PET, *ex vivo* autoradiography and histological staining for myelin (Fig. 6C). For one animal, we performed serial autoradiography and staining throughout the whole cerebellum (Sup. Fig. 2**)**. As shown in this figure, it is possible to clearly delineate where the lesion starts and finishes based on the autoradiographic signal.

### PET imaging in healthy Rhesus macaques

After confirming that the tracer can detect demyelinated lesions in rodents and to assess the translatability of the tracer to humans, we performed whole body and focused brain imaging on four healthy Rhesus macaques.

**Figure 7:**
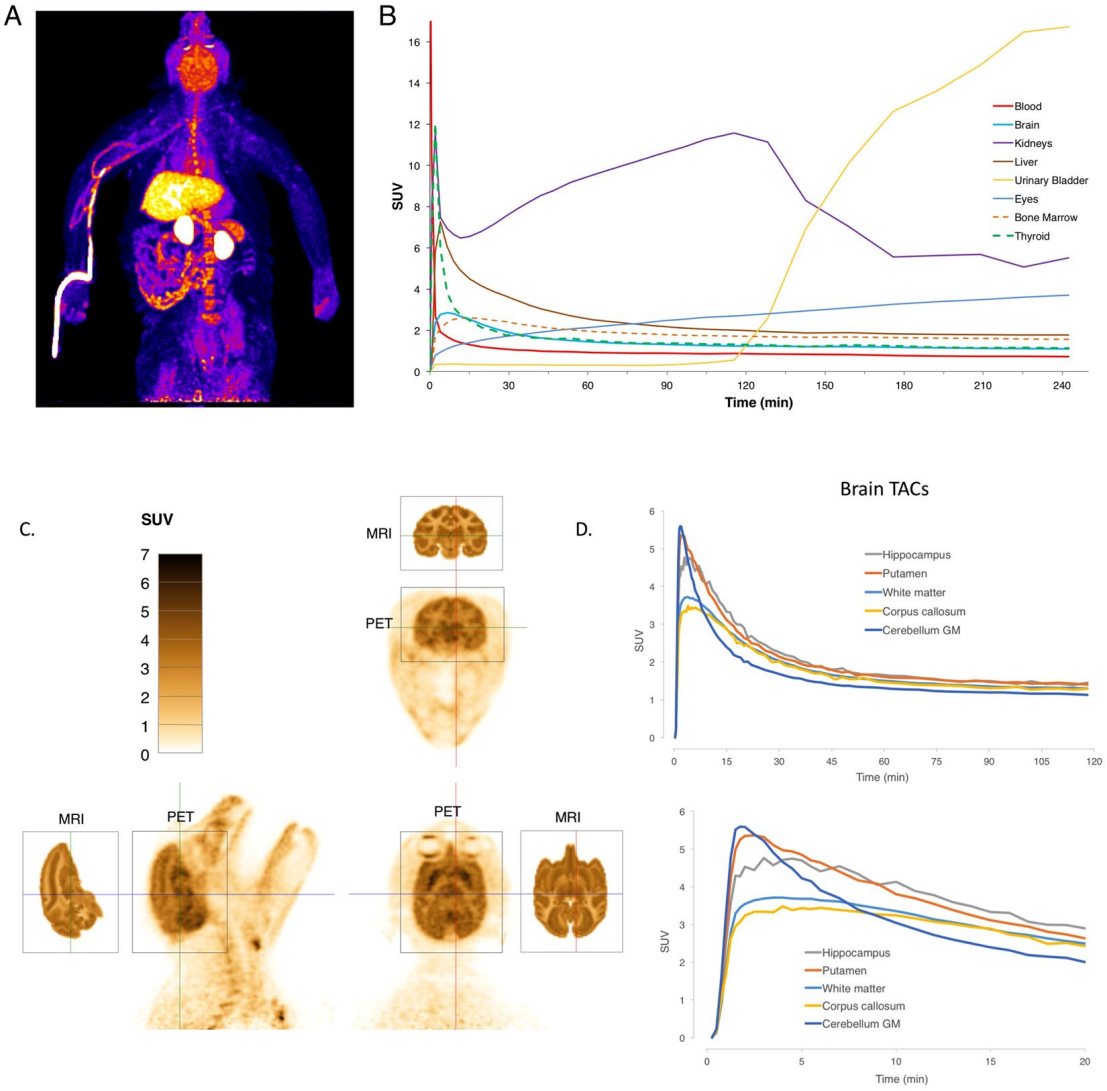
Characterization of [^18^F]3-F-4-AP in Rhesus monkeys. (**A**) Maximum intensity projection of [^18^F]3-F-4-AP from 2–6 min post injection of one monkey. (**B**) Time activity curves for major organs decay-corrected for time of injection for the same monkey. (**C**) High resolution brain images from a second monkey (2–6 min). Corresponding MRI slices from NeuroMaps atlas are included for reference. (**D**) Corresponding decay-corrected time activity curves (0–120 min and 0–20 min) for selected brain regions.

Immediately after intravenous administration of the radiotracer, radioactivity was seen in the lungs and circulatory system. Within a few minutes, the drug localized to the liver, kidney, brain, bone marrow, and thyroid and pituitary glands. Gradually, the signal in the liver, brain and glands decreased, while the signal in the kidneys, gallbladder, urinary bladder, eyes, soft tissue and skin increased (Fig. 7A and B and Sup. movies 1 and 2). Interestingly, the signal in the kidneys did not decrease continuously, as typically seen with small molecule radiotracers. Instead, there was accumulation for the first two hours, after which the signal transferred quickly into the urinary bladder (Sup. Fig. 3). The kidneys contain large numbers of K^+^ channels, and we hypothesize that the retention in the kidneys is due to the drug binding to these channels as it is cleared. In addition, the eyes accumulate radioactivity over time. It is known that many small molecule drugs bind to melanin in the eyes^70^, and this appears to be the case for [^18^F]3-F-4-AP. This is strongly supported by the fact that we saw eye signal only in pigmented rats but not in albino rats (Sup. Fig. 4). Furthermore, the radioactivity in the monkey eyes, which have about ten times more melanin than humans, appeared to localize to the iris and the retinal pigmented epithelium, where the concentration of melanin is highest^70^ (Sup. Fig. 5 and Sup. movie 3). Unlike the signal in the brain, the time-activity curves for the eyes showed a non-saturable increase, suggesting non-specific binding.

Focused high resolution brain imaging showed heterogeneous signal across the brain with maximum signal in the hippocampus and putamen and minimum signal in white matter areas such as the corpus callosum. Comparison of the PET images with MRI images from a Rhesus atlas^71^ clarifies the primary tracer primary localization to gray matter, as we previously saw in rodent autoradiography. Time-activity curves for selected brain regions show fast entry into the brain (peak SUV: 3.2–5.5 at 2–5 min) and slow to moderate washout (Fig. 7D). In some areas, such as the cerebellar cortex, the signal starts very high but decreases faster than in other areas. It is unlikely that these regional differences are mainly due to blood perfusion since some regional differences persist even after 30 min (Sup. Fig. 6).

In order to quantify specific binding in the Rhesus brain, we attempted to block the signal with cold drug (self-block). Given the prior findings in rats and to minimize the risk of causing seizures to the monkeys, we limited the dose to 0.1 mg/kg (we estimated the seizure dose in monkeys to be 1.5–3.0 mg/kg based on the rodent data). At this dose, no changes in brain signal or time activity curves were detected (Sup. Fig. 7). However, the lack of physiological effects suggests that we were well under saturation of the receptor in these experiments.

## Discussion

In addition to its central role in multiple sclerosis, demyelination is emerging as an important pathological feature in many neurological conditions, including traumatic brain injury, psychiatric disorders, and Alzheimer’s disease. In order to fully understand the role that demyelination plays in these and other neurological conditions, accurate noninvasive imaging methods to measure changes in myelination are pressingly needed. Currently, MRI is the gold standard for imaging myelin disorders. Several advanced MRI methods, such as myelin water fraction, magnetization transfer ratio, and diffusion tensor imaging have been proposed to correlate with changes in myelin^72–74^. Nevertheless, it is still challenging to discriminate between processes that often coexist –such as demyelination, remyelination, inflammation and axonal loss– based solely on MRI. On the other hand, PET has the ability to provide specific biochemical information to complement MRI findings. Given the high sensitivity of PET scanners, PET can provide information associated with biochemical changes well before macroscopic changes have occurred, potentially enabling early diagnoses and accurate assessment of demyelination and remyelination. This latter ability is particularly timely given the recent focus on developing therapies that promote remyelination^75–77^. PET is therefore a potentially useful diagnostic tool for assessing changes in MS^78^, but its use is currently limited, among other reasons, by the lack of tracers that positively correlate with demyelination.

In the recent years, it has been found that PET tracers originally developed for amyloid also bind to myelin leading to their use for imaging changes in myelination^17–19,21,22^. For example, [^11^C]PIB has been used in humans to detect demyelination and remyelination^20,79^. Nevertheless, two important limitations exist with these tracers: first, it appears that binding to myelin is non-specific and non-saturable. Specific and saturable binding is typically required for reliable quantification of receptor density by PET. In addition, with these tracers, demyelination correlates with decreases in signal rather than increases, which makes it difficult to detect small demyelinated lesions due to spillover signal from adjacent areas or partial-volume effects. In order to develop a tracer that positively correlates with demyelination, we postulated that proteins exposed in demyelinated axons would be useful targets. Voltage-gated K^+^ channels K_v_1.1 and K_v_1.2 are two examples of such proteins. Normally, these channels localize in the juxtaparanodal region of axons beneath the myelin sheath. Following demyelination, potassium channels become exposed, migrate throughout the demyelinated segment and increase in expression^25,42,80^. The MS drug 4-AP binds to these channels and enhances conduction: based on this property we hypothesized that 4-AP could serve as the foundation for a tracer for demyelination.

In this report, we describe the distribution of [^14^C]4-AP after *in vivo* administration in the brains of normal mice and several models of myelin abnormalities probed by autoradiography. Carbon-14 autoradiography is an excellent tool for early validation of new tracers as it does not require synthesizing the tracer before each use. Its virtually endless half-life (5,730 years) allows indefinite storage of compounds, making many ^14^C-labeled drugs commercially available. Furthermore, ^14^C-labeled drugs can be synthesized with molar activities near their theoretical maximum, indicating that the vast majority of the injected molecules are actually radiolabeled. In addition, film autoradiography offers over a hundred-fold better resolution than PET (~10 μm *vs*. >1 mm) making it ideal to study sub-organ distribution in small laboratory animals like mice. In control mice, [^14^C]4-AP shows high binding in non-myelinated areas (*i.e*., gray matter) and low binding in myelinated areas (*i.e*., white matter) (Fig. 1A). In a mouse model of dysmyelination (Shiverer) and two models of demyelination (LPC and DTA), we detected a high level of binding in areas of demyelination (Fig. 1B,C) supporting the hypothesis that 4-AP does not bind to white matter areas where the K^+^ channels are covered by myelin. Following remyelination (DTA model), [^14^C]4-AP binding decreases (Fig. 1C), suggesting that 4-AP and derivatives could potentially serve as reporters for CNS remyelination.

Following these observations, we decided to develop an analog of 4-AP that could be labeled with ^18^F and serve as a PET tracer to quantify changes in myelination. We synthesized four fluorinated derivatives of 4-AP (Fig. 2A) and found one compound, 3-F-4-AP, that has similar binding affinity to the channel as 4-AP (Fig. 2B–G), can cross the BBB, is metabolically stable, is active *in vivo*, and has fast pharmacokinetics (Fig. 3A,B). We then showed by *ex vivo* autoradiography that [^14^C]3-F-4-AP has high uptake in demyelinated areas and can be used to distinguish demyelinated animals from controls (Fig. 4A–C). When we labeled this molecule with ^18^F and conducted PET imaging experiments in healthy rats, we saw localization of radioactivity in myelin-poor areas, which was confirmed by ^18^F-autoradiography (Fig. 5C,D). Furthermore, PET imaging of demyelinated rats (LPC model) showed higher binding of [^18^F]3-F-4-AP in demyelinated areas than control areas (Fig. 6A). The difference in SUV between the LPC-lesioned side of the cerebellum and the contralateral side was sufficient to distinguish demyelinated animals from controls (Fig. 6B), and there was excellent correlation between PET, autoradiography, and LFB staining (Fig. 6C and Sup. Fig. 2). All of the rodent CNS demyelination models used here present with minimal or no inflammation and disruption of the BBB^41,45,46^. These processes are present in MS (1) as well as in other preclinical models such as experimental autoimmune encephalomyelitis (EAE)^81^. Thus, an important next step will be to examine [^18^F]3-F-4-AP in EAE models. Since [^18^F]3-F-4-AP readily crosses the BBB, we do not expect that changes in the BBB will have a major impact on the PET signal associated with CNS demyelination. Nevertheless, since microglial cells and T cells also express K_v_1.3 channels^82^, it will be important to study the possible effect that inflammation may have on the [^18^F]3-F-4-AP PET signal.

Further evaluation of the radioligand in Rhesus macaques showed that the radioligand has excellent properties for brain imaging. The tracer shows fast entry into the brain and slow to moderate washout (Fig. 7). The SUV for the whole brain peaks within the first five minutes to a value around 4. Within the brain, the highest binding is in gray matter and the lowest in white matter, which is consistent with the hypothesis and correlates well with the rodent PET and autoradiography findings. In addition, [^18^F]3-F-4-AP is not defluorinated *in vivo* as evidenced by the lack of bone uptake.

Although we could not perform blocking studies by PET or autoradiography to quantify specific binding due to the risk of causing seizures to the animals, all evidence points to a high degree of specific binding to K^+^ channels: first, non-specific binding would be expected to occur in postmortem tissue but when brain tissue is incubated with [^14^C]4-AP or [^14^C]3-F-4-AP *in vitro* no binding is observed; second, the binding in demyelinated areas in rodent brains cannot be explained by changes in BBB permeability because 3-F-4-AP is BBB permeable and the BBB remains intact in the models studied; third, non-specific binding typically arises from hydrophobic interactions with brain lipids in white matter but 3-F-4-AP has lowest binding in white matter; fourth, the time-activity curves across brain regions are consistent with high specific binding as non-specific binding would be expected to increase over time (as seen in the eyes); fifth, the unusual retention in the kidneys is likely due to binding to K^+^ channels in those organs; and, sixth, the pharmacological effects of 3-F-4-AP and 4-AP (*i.e*., seizures) are best explained by direct binding to K^+^ channels in the CNS.

In summary, this report shows, as proof of principle, that voltage-gated K^+^ channels can serve as targets for brain imaging. More specifically, [^18^F]3-F-4-AP can be used to image K_v_ channels by PET. These channels, which have not been previously imaged by PET, are implicated in many pathologies and are important therapeutic targets^83^. Notably, the signal of [^18^F]3-F-4-AP increases with demyelination, a highly useful imaging property that may enable quantitative evaluation of new therapies currently being developed to reverse demyelination^44,76,84–90^.

## Methods

Experiments involving animals were performed in accordance with relevant guidelines and regulations. All rodent procedures were approved by the Institutional Animal Care and Use Committees of the University of Chicago. All monkey procedures were approved by the Institutional Animal Care and Use Committee of the National Institutes of Health Clinical Center. 4-AP derivatives have similar proconvulsant properties as 4-AP and must be handled with caution. All the procedures involving radioactivity were performed by trained personnel and approved by the corresponding radiation safety office.

### Mouse and rat strains

8–10 week-old Shiverer mice, *Mbp*^*shi/shi*^, (male and female) were obtained from the Jackson Laboratory (Stock number: 001428), bred in-house and genotyped. Wild-type littermates, *Mbp*^+/+^, were used as controls. The DTA model was developed in the laboratory^45^ and is a cross between B6.Cg-Tg(Plp1-cre/ERT)16Pop (Jackson Laboratory Stock number: 005975) mice, which have a tamoxifen inducible Cre-mediated recombination system driven by the mouse Plp1, proteolipid protein (myelin) 1 promoter, and Gt(ROSA)26Sor^tm1(DTA)Jpmb^/J (Jackson Laboratory Stock number: 006331) mice in which Diphtheria toxin fragment A (DTA) transcription is prevented until crossed to a strain expressing Cre recombinase under the control of a promoter of interest, resulting in ablation of Cre-expressing cells. 8-week-old female DTA mice were injected with 0.8 mg of 4-hydroxytamoxifen daily for three consecutive days. The 4-hydroxytamoxifen was dissolved in DMSO:ethanol:oil (4:6:90) mixture at a concentration of 8 mg/mL. Mice were housed in cages of 4–5 under a 12 h light/dark cycle. For the PET/CT studies 8–12 week-old male Sprague Dawley and Long Evans rats were used.

### Monkeys

Four male Rhesus monkeys (macaca mulatta) 8–12 years-old were used in the study. Their weighs ranged from 8.8 to 13 kg.

### Lysolecithin induced demyelination in mice and rats

This procedure was conducted as previously described^19^. See Supporting Information (SI) for additional details.

### ^14^C-labeled drugs and concentrations

[2,6-^14^C]4-aminopyridine (4-AP) and [2,6-^14^C]4-amino-3-fluoropyridine (3-F-4-AP) were synthesized by American Radioactive Chemicals (100 mCi/mmol). These drugs were dissolved in sterile saline to a final activity of 4 μCi/100 μL for 4-AP and 3 μCi/100 μL for 3-F-4-AP. [1-^14^C]2-deoxy-D-glucose (45–60 mCi/mmol) was acquired from Perkin Elmer and dissolved in saline to a final activity of 1 μCi/100 μL.

### *Ex vivo* autoradiography

^14^C labeled drugs (4-AP, 3-F-4-AP, 2-DG) were injected into awake mice (single drug per animal) via tail vein (200 μL for a 20 g mouse) while temporarily restrained. Number of animals per group was determined using statistical power analysis (α = 0.05, power = 0.80). The radioactivity administered was 0.4 μCi/g for 4-AP, 0.3 μCi/g for 3-F-4-AP and 0.1 μCi/g for 2-DG. After injection, the animal was returned to its cage and allowed to walk around for 30 min (3-F-4-AP) or 45 min (4-AP and 2-DG), after that time the animal was euthanized and their brains dissected. The brains were embedded in Optimal Cutting Temperature (Tissue-Tek), frozen in dry ice and stored at −80 °C. Frozen brains were sliced in a cryostat at 20 μm thickness, the sections mounted on Superfrost Plus slides (Fisher) and allowed to dry at room temperature for at least 30 min. Next, the slides were placed in a cassette in front of autoradiography film (Denville scientific) sealed in the dark at −80 °C for 14–21 days. After that time the cassette was allowed to warm up to room temperature and the films developed inside a dark room using a film processor (Konica). The films were then scanned using an EPSON Perfection V750 scanner (Positive film mode, 8-bit grayscale, 1,200 dpi). After digitizing the radiographs, the same slides were stained using Luxol fast blue (LFB) and cresyl violet. For [^18^F]3-F-4-AP autoradiography in rats we injected 1–2 μCi per g. After euthanasia, the brains were dissected without perfusion, frozen in dry ice and sliced in a cryostat at −20 °C.

### Histochemistry

Slides were stained with Luxol Fast Blue (myelin) and cresyl violet (nuclear). See SI for additional details.

### Expression of Shaker K^+^ channel in Xenopus laevis oocytes and recording of K^+^ currents

K^+^ channel expression^53^ and current recording^51^ were performed as previously described. See SI for additional details.

### Dissection of optic nerves and electrophysiology

Optic nerves were dissected from 12–16 week old Shiverer (Mbp^*shi/shi*^) and control mice (*Mbp*^+*/shi*^ and *Mbp*^+*/*+^). Compound action potentials (CAP) were recorded using suction electrodes as described by Stys *et al.*^91^. See SI for additional details.

### Electrophysiology data analysis

The Hill equation used to fit the data from the voltage-clamp and the optic nerve experiments was as follows: y = y_0_ + (y_f_ − y_0_) * x^n^/(k^n^ + x^n^); where n refers to the Hill coefficient (typically 1.0 ± 0.1) and k refers to EC_50_ or IC_50_. y_0_ and y_f_ refers to the origin and final ordinate values and were constrained to 1.0 ± 0.1 or 0.0 ± 0.1 depending on the experiment. EC_50_ = 10^<logEC50>^; where <logEC_50_>  = average of logEC_50_ from all experiments with the same drug. CI_95_ = *[Upper Limit*. *Lower Limit]*; where *Upper Limit* = 10^(logEC50+s.d.)^ and *Lower Limit* = 10^(logEC50−s.d.)^.

### Partition coefficient (logD)

The partition coefficient octanol-water at pH 7.4 was measured by adding 0.1 mg of the compound to a 10 mL 50:50 mixture of octanol:PBS. The vial was shaken vigorously followed by 1 min centrifugation at 1,000 g to separate the phases. The drug concentration in each phase of the drug was calculated from the area under the peak on reverse phase HPLC (10 μL sample) using a calibration curve.

### *In vivo* effects

10-week-old female C57Bl/6 J mice were given an intraperitoneal injection of the drug under investigation and monitored continuously for 4 h. After 4 h no signs of drug effects could be observed. At least 72 h passed between injections to the same mice.

### Parallel artificial membrane permeability assay (PAMPA) and Stability in plasma and microsomes

These experiments were performed by a Contract Research Organization (Selvita, Inc.) following published procedures^92^.

### Radiochemical synthesis of [^18^F]3F4AP studies and quality control procedures

Radiochemical synthesis for rat studies was performed as described by Brugarolas *et al.*^66,67^. Radiochemical synthesis for monkey studies was performed as described by Basuli *et al.*^68^.

### Organ distribution

50–60 uCi of the [^18^F]3-F-4-AP tracer was injected via tail vein to eight 8-week-old female B6 albino mice. At the specified times, the mice were euthanized by anesthetic overdose. Following euthanasia blood was collected by cardiac puncture and transferred to EDTA coated tubes. Other organs (brain, liver and kidneys) were collected by dissection and transferred to glass tubes. Radioactivity was measured using an automatic gamma counter (^18^F protocol) and corrected for organ weight and injected dose. Using the original tracer solution, 8 standard solutions ranging from 20 μCi to 10^−5^ μCi were prepared, analyzed by gamma counting and used as a calibration curve to correlate peak area of the samples to concentration. SUV was calculated as SUV = (dose organ/organ weight)/(dose injected/body weight) with dose corrected for decay.

### PET/CT imaging in rats

PET/CT imaging studies were carried out on a Trifoil Triumph™ Trimodality Preclinical microPET/SPECT/CT Imaging System (Norridge, CA) equipped with a X-PET detector constructed from BGO crystals. Rats were anesthetized with a mixture of 2% isoflurane and 4% oxygen and kept under anesthesia throughout the whole procedure. A CT image was acquired first (at 40 kV, 140-uA beam current, and 256 projections, approx. 5 min). After the CT, 50–150 µCi of [^18^F]3-F-4-AP was infused as a bolus through a tail vein injection port and a 25, 30 or 90 min list-mode PET scan performed. PET data are acquired using a sensitivity 5.9%, 250- to 750-keV energy window and 12-ns timing window in list-mode format with spatial resolution of 1.6–2.0 mm (FWHM), which can be binned into 3-dimensional (3D) sinograms corresponding to total and true events, respectively. Images were reconstructed using Fourier rebinning followed by a 2-dimensional ordered-subsets expectation maximization algorithm (OSEM) (4 iterations with 15 subsets). Reconstructed images were processed by using VivoQuant (inviCRO, LLC, Boston) version 2.1. Time activity curves were generated by processing the PET data in 5 min frames.

### Detection of demyelination by PET

A power analysis (power 0.8, α = 0.05) based on the autoradiography results was used to determine the number of rats needed to detect a statistically significant change in signal. Randomly selected rats were injected with lysolecithin or saline intracranially as described above. 0–25 min dynamic PET scan was performed 6–8 days post intracranial injection (peak of demyelination) after administration of 0.3–0.5 μCi/g. Within 24 h of the PET scan, the rats received a second dose of tracer (1–2 uCi/g), the animals were sacrificed 25 min post injection and their brains processed for autoradiography as described above. Image analyst was not blind to the treatment group. Post autoradiography, LFB staining was performed to assess demyelination. Animals showing physical damage to the brain due to the injection were excluded from the study. Brain ROI analysis was performed using reference atlas with VivoQuant version 2.1 (inviCRO, LLC, Boston).

### Whole body PET/CT imaging in Rhesus macaques

Whole body PET/CT imaging was carried out on a Siemens mCT PET/CT. Monkeys were sedated with ketamine and intubated. Following intubation monkeys were placed in prone position on the scanner bed face forward with their head immobilized in a stereotaxic frame. The animals were then anesthetized with 1% isoflurane and kept under anesthesia throughout the whole procedure. A CT image was acquired first (approx. 5 min). After the CT, 5 mCi in 8 mL of saline of [^18^F]3-F-4-AP was infused as a bolus through the cubital vein. Scans were obtained in list mode and reconstructed with a 3D ordinary Poisson OSEM algorithm as 30 frames of data (2 × 15 secs, 4 × 30 secs, 8 × 60 secs, 8 × 120 secs, 8 × 240 secs). All activities were decay-corrected to the time of injection of the radiopharmaceutical. Reconstructed images were processed using VivoQuant. ROIs for the major organs were hand drawn based on the CT and used to calculate time-activity curves.

Focused high-resolution brain imaging in Rhesus macaques: brain PET imaging was carried out on a Siemens HRRT PET scanner. Monkeys were sedated with ketamine and intubated. Following intubation monkeys were placed in prone position on the scanner bed face forward with their head immobilized in a stereotaxic frame. The animals were then anesthetized with 1% isoflurane and kept under anesthesia throughout the whole procedure. Monkeys received 5 mCi of [^18^F]3-F-4-AP as a bolus through the cubital vein. Scans were obtained in list mode and reconstructed with a 3D ordinary Poisson OSEM algorithm as 30 frames of data (8 × 15 secs, 8 × 30 secs, 16 × 60 secs, 16 × 120 secs, 16 × 240 secs). All activities were decay-corrected to the time of injection of the radiopharmaceutical. Reconstructed images were processed using VivoQuant. The Rhesus brain atlas from VivoQuant was fitted to the data and used to calculate time-activity curves for 43 different brain regions.

## Electronic supplementary material

Supplemental movie 1

Supplemental movie 2

Supplemental movie 3

Supplemental Information
