# Supplementary material for: Development of a PET radioligand for potassium channels to image CNS demyelination
Source: Sci Rep. 2018 Jan 12;8:607. doi: 10.1038/s41598-017-18747-3 (PMC5766510; doi:10.1038/s41598-017-18747-3)
Supplement: Supplementary file 4 — Supplemental Information [file 41598_2017_18747_MOESM4_ESM.pdf]

Development of a PET radioligand for potassium channels to image CNS demyelination

Authors: Pedro Brugarolas[1a]*, Jorge E. Sánchez-Rodríguez[2b], Hsiu-Ming Tsai[3], Falguni Basuli[4], Shih-Hsun Cheng[3], Xiang Zhang[4], Andrew V. Caprariello[5c], Jerome J. Lacroix[2d], Richard Freifelder[3], Dhanabalan Murali[6], Onofre DeJesus[6], Robert H. Miller[5e], Rolf E. Swenson[4], Chin-Tu Chen[3], Peter Herscovitch[7], Daniel S. Reich[8], Francisco Bezanilla[2], Brian Popko[1]*

Affiliations:

[1] Department of Neurology, University of Chicago, Chicago, IL

[2] Department of Biochemistry and Molecular Biology, University of Chicago, Chicago, IL

[3] Department of Radiology, University of Chicago, Chicago, IL

[4] Imaging Probe Development Center, NIH/NHLBI, Bethesda, MD

[5] Department of Neurosciences, Case Western Reserve University, Cleveland, OH

[6] Department of Medical Physics, University of Wisconsin at Madison, Madison, WI

[7] Positron Emission Tomography Department, NIH/CC, Bethesda, MD

[8] Translational Neuroradiology Section, NIH/NINDS, Bethesda, MD

Author present addresses:

[a] Massachusetts General Hospital, Boston, MA

[b] Universidad de Guadalajara, Guadalajara, Jalisco, Mexico.

[c] University of Calgary, Calgary, Alberta, Canada.

[d] Western University of Health Sciences, Pomona, CA

[e] George Washington University, Washington, DC

*To whom correspondence should be addressed:

Pedro Brugarolas, Ph.D.	Tel. (617) 643-4574	E-mail: pbrugarolas@mgh.harvard.edu
Brian Popko, Ph.D.	Tel. (773) 702-4953	E-mail: bpopko@uchicago.edu

PAGE ... CONTENTS

4. **Supplemental methods:** Chemical syntheses of 4-AP derivatives

10. **Supplemental figure 1**. [^{14}C]4-AP autoradiography of demyelinated spinal cords.

11. **Supplemental Figure 2**. Serial autoradiography and LFB images of the cerebellum of a demyelinated rat.

12. **Supplemental Figure 3**. Time series images and time-activity curves of kidney and bladder.

13. **Supplemental figure 4**. Eye uptake in gray and albino rat

14. **Supplemental figure 5**. Monkey eye distribution

15. **Supplemental figure 6**. Time dependent PET images of the Rhesus brain

16. **Supplemental figure 7**. Brain TACs after preinjection of 0.1 mg/kg of [^{18}F]3-F-4-AP

17. **Supplemental movie 1.** Rotating maximum intensity projection of a whole-body monkey scan (2-6 min post injection).

18. **Supplemental movie 2.** Dynamic PET images time series. Each frame represents 3 one cycle through 3 bed positions (2 × 15 secs, 4 × 30 secs, 8 × 60 secs, 8 × 120 secs, 8 × 240 secs).

19. **Supplemental movie 3.** Rotating view of the monkey eyes and forehead.

SPECTRAL DATA:

20. ^{1}H NMR tert-butyl N-[3-(fluoromethyl)pyridin-4-yl]carbamate **(9)**

21. ^{13}C NMR tert-butyl N-[3-(fluoromethyl)pyridin-4-yl]carbamate **(9)**

22. ^{19}F NMR tert-butyl N-[3-(fluoromethyl)pyridin-4-yl]carbamate **(9)**

23. HRMS tert-butyl N-[3-(fluoromethyl)pyridin-4-yl]carbamate **(9)**

24. ^{1}H NMR 3-fluoromethyl-4-aminopyridine (**5**)

25. ^{19}F NMR 3-fluoromethyl-4-aminopyridine (**5**)

26. HRMS 3-fluoromethyl-4-aminopyridine (**5**)

27. ^{1}H NMR tert-butyl N-[3-(2-hydroxyethyl)pyridin-4-yl]carbamate (**11**)

28. ^{1}H NMR tert-butyl N-[3-(2-fluoroethyl)pyridin-4-yl]carbamate (**12**)

29. ^{13}C NMR tert-butyl N-[3-(2-fluoroethyl)pyridin-4-yl]carbamate (**12**)

30. ^{19}F NMR tert-butyl N-[3-(2-fluoroethyl)pyridin-4-yl]carbamate (**12**)

31. HRMS tert-butyl N-[3-(2-fluoroethyl)pyridin-4-yl]carbamate (**12**)

32. ^{1}H NMR 3-fluoroethyl-4-aminopyridine (**6**)

33. ^{13}C NMR 3-fluoroethyl-4-aminopyridine (**6**)

34. ^{19}F NMR 3-fluoroethyl-4-aminopyridine (**6**)

35. HRMS 3-fluoroethyl-4-aminopyridine (**6**)

Supplemental Methods

Chemical syntheses of 4-AP derivatives: All chemicals were ordered from Sigma unless otherwise noted.

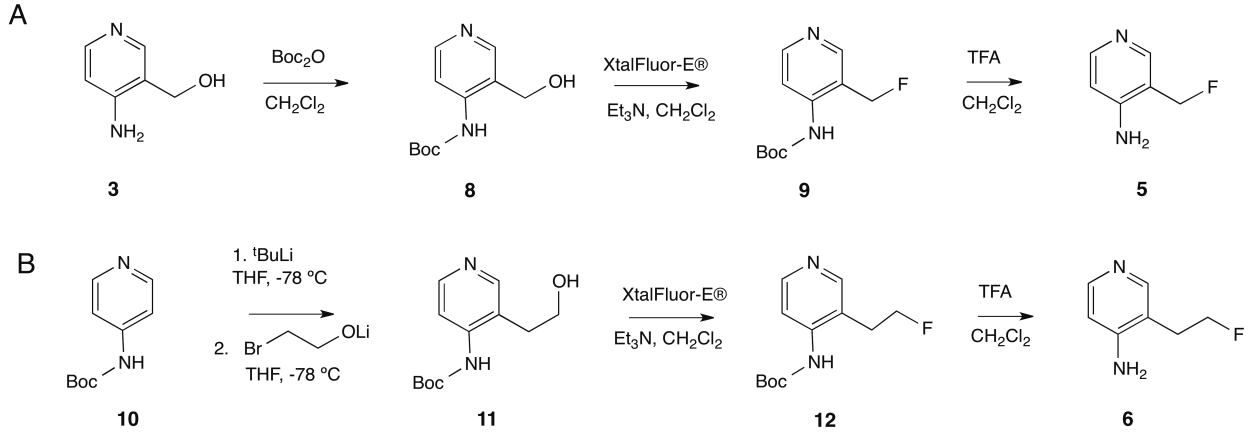

tert-butyl N-[3-(hydroxymethyl) pyridin-4-yl] carbamate **(8):** To a solution of 4-aminopyridine-3-methanol (**3**) (Alfa Aesar) (806 mg, 6.5 mmol) in CH_2Cl_2 (10 mL) a solution of di-*tert*-butyl-dicarbonate (1.43 g, 6.56 mmol) in CH_2Cl_2 (5 mL) was added and stirred at room temperature for 1 h (TLC). After 1 h, the solution was acidified with 1 N HCl (7.4 mL, 7.4 mmol). The phases were separated and the aqueous phase was washed with CH_2Cl_2. The aqueous layer was mixed with a fresh portion of CH_2Cl_2 (10 mL) and treated with K_2CO_3 (711 mg, 5.1 mmol). The phases were separated and treated with additional amounts of CH_2Cl_2.. The combined organic extracts were dried ($MgSO_4$) and evaporated *in vacuo* to give a 60:40 mixture containing desired product **8** and the O-linked carbonate. Attempts to isolate the N-carbamate by flash chromatography from the O-carbonate were unsuccessful due interconversion between these two species in solution at room temperature and the mixture of compounds was used for the next step. ^{1}H-NMR ($CDCl_3$, 500 MHz) δ: 1.53 (9H, s), 4.67 (2H, s), 4.83 (2H, br s), 7.95 (1H, s), 8.07 (1H, d, J = 5.5 Hz), 8.28 (1H, d, J = 5.5 Hz), 8.48 (1 H, s). This product has been previously synthesized through a different route(Mochizuki et al., 2011). Spectral data for this and other compounds can be found in the supplementary information.

tert-butyl N-[3-(fluoromethyl)pyridin-4-yl] carbamate **(9):** To a solution of triethylamine (450 µL, 2.76 mmol) in CH_2Cl_2 (5 mL) at – 78 °C was added XtalFluor-E® (473 mg, 207 mmol) and the product from the previous reaction (310 mg, 1.38 mmol in 5 mL CH_2Cl_2). The reaction was stirred at 0 °C for 15 min (TLC). Subsequently, the reaction mixture was washed with $NaHCO_3$ (10 mL) and

brine (10 mL). The organic phase was dried ($MgSO_4$) and concentrated *in vacuo*. The crude product was purified by flash chromatography to afford **9** (113 mg, 36 % yield). R_f = 0.5 (1:1, hexanes: EtOAc). ^{1}H-NMR ($CDCl_3$, 500 MHz) δ (ppm): 1.56 (9H, s), 5.48 (2H, d, J = 48 Hz), 7.12 (1H, br s), 8.12 (1H, d, J = 5.5 Hz), 8.38 (1H, d, J = 3 Hz), 8.32 (1H, s), 8.53 (1H, dd, J_2 = 5.5 Hz, J_1 = 1.0 Hz). ^{13}C-NMR ($CDCl_3$, 125 MHz) δ: 28.2, 80.6, 82.0 (d, J = 15.5 Hz), 113.2, 118.0 (d, J = 15.5 Hz), 145.6, 150.1, 149.7, 151.7, 152.4. ^{19}F-NMR ($CDCl_3$, 470 MHz) δ: -209.3 (t, J = 48 Hz). HR-MS *m/z*: 227.1190 $(M+H)^+$.

3-fluoromethyl-4-aminopyridine **(5):** To a solution of 9 (56 mg, 0.25 mmol) in CH_2Cl_2 (3 mL) was added TFA (194 µL, 2.5 mmol) at 0 °C and stirred at room temperature for 5 h (TLC). After 5 h the reaction was quenched with excess NaOH (1 M). The solvent was evaporated to afford **5** quantitatively. R_f = 0.2 (MeOH). ^{1}H-NMR (500 MHz, D_2O) δ (ppm): 5.40 (2H, d, J = 48 Hz), 6.87 (1H, d, J = 7 Hz), 7.95 (1H, d, J = 7 Hz), 8.09 (1H, s). ^{19}F-NMR ($CDCl_3$, 470 MHz) δ: -215.9 (t, J = 48 Hz). HR-MS *m/z*: 127.0666 $(M+H)^+$. Purity was found to be over 95% by HPLC.

tert-butyl N-[3-(2-hydroxyethyl)pyridin-4-yl] carbamate **(11):** Adapted from Spivey *et al* (Spivey et al., 1999). To a solution of 4-(Boc-amino)pyridine (**1**) (2.0 g, 10.3 mmol, 1 eq.) in 25 mL of dry THF at -78 ºC was added *t*-BuLi (14.5 mL, 1.7 M, 24.7 mmol, 2.4 eq.) in pentane over 30 min. The resulting bright yellow suspension was stirred at -78 ºC for 15 min, at -15 ºC for 2h and then recooled to -78 °C. In a separate flask, n-BuLi (7.38 mL, 2.5M, 18.54 mmol, 1.8 eq) in hexanes was added to a solution of 2-bromoethanol (1.294 mL, 15.45 mmol, 1.5 eq) in 20 mL of dry THF at -78 ºC and stirred for 10 min. After 10 min, the bromoethanol solution was transferred via cannula to the flask containing lithiated N-Boc-4-aminopyridine over 10 min. The reaction was allowed to warm to room temperature and the mixture was stirred for 2h. The reaction was recooled to -78 ºC and quenched with 5 mL of water. The solution was partitioned between water (20 mL) and CH_2Cl_2 (30 mL). The phases were separated and the extraction was completed with additional portions of CH_2Cl_2. The combined organic extracts were washed with brine, dried over $MgSO_4$ and evaporated under vacuum. The crude product was dissolved in a small amount of CH_2Cl_2 purified by silica gel chromatography (EtOAc) to afford the product **11** (0.678 g, 36% yield). R_f = 0.15 (EtOAc). ^{1}H-NMR (500 MHz, $CDCl_3$) δ (ppm): 1.51 (9H, s), 2.81 (2H, t, J = 5.0 Hz), 3.80 (1H, br s), 3.94 (2H, t, J = 5.0 Hz), 7.92 (1H, d, J = 5.5 Hz), 8.15 (1H, s), 8.27 (1H, d, J = 5.5 Hz), 8.63 (1H, s).

tert-butyl N-[3-(2-fluoroethyl)pyridin-4-yl] carbamate **(12):** To a solution of Et_3N•3HF (715 µL, 4.39 mmol, 2 eq.) in 5 mL of dry CH_2Cl_2 at 0 °C, XtalFluor E® (753 mg, 3.29 mmol, 1.5 eq) was

added and stirred for 5 min. After 5 min, 2 (525 mg, 2.195 mmol, 1 eq) was added and the reaction was monitored by TLC (1:1, hexanes:EtOAc). 15 min later the reaction was washed with $NaHCO_3$ (5 mL) and brine (5 mL), dried with $MgSO_4$ and the solvent evaporated under vacuum. The crude product was dissolved in a small amount of CH_2Cl_2 and purified by silica gel chromatography to afford **3** (457 mg, 71 % yield). R_f = 0.4 (1:1, hexanes:EtOAc). Mp = 103 ºC. ^{1}H-NMR (500 MHz, $CDCl_3$) δ (ppm): 1.53 (9H, s), 2.98 (2H, dt, J_2 = 29 Hz, J_1 = 5.8 Hz), 4.72 (2H, dt, J_2 = 47 Hz, J_1 = 5.8 Hz,), 7.04 (1H, d, J = 7.5 Hz), 7.99 (1H, d, J = 5.0 Hz), 8.32 (1H, s), 8.40 (1H, d, J = 5.0 Hz). ^{13}C-NMR ($CDCl_3$, 125 MHz) δ (ppm): 28.2, 30.4 (d, J = 20.1 Hz), 81.6, 84.9 (d, J = 165 Hz), 113.7, 121.0, 144.7, 149.7, 151.2. ^{19}F-NMR ($CDCl_3$, 470 MHz) δ (ppm): -213.3 (tt, J_2 = 47 Hz, J_1 = 29 Hz). HR-MS *m/z*: 241.1347 $(M+H)^+$.

3-fluoroethyl-4-aminopyridine **(6):** To a solution of 12 (120 mg, 0.5 mmol, 1 eq.) in 5 mL of CH_2Cl_2 was added TFA (191 μL, 2.5 mmol, 5 eq) at 0 °C. The reaction was allowed to warm up to room temperature and stirred for 5 h (TLC). After 5 h, the reaction was quenched with excess NaOH (1 M) and extracted multiple times with CH_2Cl_2. The solvent was evaporated to afford **6** quantitatively. R_f = 0.5 (MeOH). ^{1}H-NMR (500 MHz, $CDCl_3$) δ (ppm): 2.91 (2H, dt, J_2 = 26.5 Hz, J_1 = 6 Hz), 4.26 (2H, br s), 4.69 (2H, dt, J_2 = 47 Hz, J_1 = 6 Hz), 6.54 (1H, d, J = 5.5 Hz), 8.13 (1H, s), 8.15 (1H, d, J = 5.5 Hz). ^{13}C-NMR ($CDCl_3$, 125 MHz) δ (ppm): 30.2 (d, J = 20.6 Hz), 84.2 (d, J = 166 Hz), 110.1, 149.1, 151.0, 151.7. ^{19}F-NMR ($CDCl_3$, 470 MHz) δ (ppm): -213.3 (tt, J_2 = 47 Hz, J_1 = 26.5 Hz). HR-MS *m/z*: 141.0823 $(M+H)^+$. Purity was found to be over 95% by HPLC.

Lysolecithin induced demyelination in mice and rats: This procedure was conducted as previously described (Wang et al., 2009). For mice, we injected 2 μL of 2% LPC in at the following coordinates relative to bregma: -1.2 mm anterior-posterior, 1.0 mm medial-lateral, 1.0 mm superior-inferior. For rats (Sprague Dawley), we injected 3 μL of 1% LPC in at the following coordinates relative to lambda: -1.2 mm anterior-posterior, 3.0 mm medial-lateral, 4.0 mm superior-inferior.

Histochemistry: The slides were washed in PBS (3x 5 min) and then submerged for 1 h in 4% PFA for fixation. After fixing the tissue, the slides were washed in PBS (3x 5 min) rinsed in ddH_2O, and submerged in ice-cold acetone for 20 min for permeabilization. After permeabilizing the tissue, slides were rinsed in water, placed in EtOH (70 % for 1 min, 95 % for

1 min) and left in LFB solution (0.1 g LFB, 100 mL of 95% EtOH, 0.5 mL glacial acetic acid) overnight at 56 ºC. Next morning, the slides were rinsed in 95% EtOH and ddH_2O to remove the excess of dye, differentiated in lithium carbonate solution (0.05 g in 100 mL) for 30 s, 70% EtOH for 30 s and rinsed in ddH_2O. The differentiation steps were repeated until the gray matter was clear and the white matter appeared blue and sharply defined. Afterwards, the slides were counterstained in cresyl violet (0.1 g in 100 mL of ddH_2O, 0.1 mL acetic acid) for 30 s. The slides were differentiated in 95 % EtOH for 5 min and dehydrated (100% EtOH, 2x 5 min ea; 100 % xylene, 2 x, 5 min ea) before mounting with permount (Electron Microscopy Sciences). After staining, images of the slides were collected using the CRi Pannoramic Whole Slide Scanner (Perkin Elmer) equipped with a 20x objective.

Image analysis: The histology images were superimposed on the autoradiography images using Photoshop. The histology images were used to precisely outline the corpus callosum, the cortex and a ~1.5 mm-wide area around the brain (background). The autoradiographic signal from each area was quantified. The background signal was subtracted from the all the areas and defined as zero. The signal intensity of the cortex was set as 1.0 and the signal intensity of the corpus callosum was measured relative to the cortex. Color images were created using the 6-shades look up table (LUT) in ImageJ.

Expression of Shaker K^+ channel in Xenopus laevis oocytes: K^+ channel expression in *Xenopus* oocytes membranes was achieved by injecting approximately 50 ng of non-inactivating Shaker (Hoshi et al., 1990) cRNA (kit Ambion) into the oocytes 24 h after surgical extraction from adult frogs and collagenase treatment. Injected oocytes were maintained in a standard oocytes solution (100 mM NaCl, 5 mM KCl, 2 mM $CaCl_2$, and 10 mM Hepes at pH 7.5) at 16.5 °C and recordings were performed 1-3 days after injection.

Recording of K^+ currents in Xenopus laevis oocytes: K^+ currents were recorded from oocytes expressing Shaker K^+ channels using the cut-open voltage clamp technique as described by Stefani and Bezanilla (Stefani and Bezanilla, 1998). The internal solution was 120 mM KOH, 20 mM HEPES-methyl sulfonate (MES) pH 7.4, 2 mM EGTA. The external solution was 12 mM KOH, 105 mM N-methyl-D-glucamine-MES pH 7.4, 20 mM HEPES, 2 mM CaOH. To assess the effects of

the 4-AP derivatives, the drug under study was added in incremental concentrations by exchanging the external solution (top and guard chambers) several times. After application, cells were voltage-clamped at least 5 min at 0mV, then voltage-clamped at -80mV for 1-2 min. K^+ currents were generated by applying series of 50 ms pulses from -70 mV to +40 mV in increments of 10 mV. The effect of the drug was assessed by measuring the relative intensity of the K^+ current before and after applying varying drug concentration at a constant voltage (typically +20 mV) and at the end of the test-pulse. Data acquisition and analysis of the traces was performed using Gpatch software (Department of Anesthesiology, University of California, Los Angeles). The half-maximal inhibitory concentration (IC_{50}) for each drug was calculated by plotting the relative K^+ current *vs*. concentration and fitted to the Hill equation using the software Origin.

Dissection of optic nerves*:* Optic nerves were dissected from 12-16 week old Shiverer ($Mbp^{shi/shi}$) and control mice ($Mbp^{+/shi}$ and $Mbp^{+/+}$). Mice were euthanized by CO_2 overdose and the optic nerves were quickly dissected between the eyeball and the optic chiasm. The nerves were incubated for 30 min at 37 °C in oxygenated (95% O_2, 5% CO_2) aCSF solution (126 mM NaCl, 3 mM KCl, 2 mM $MgSO_4$, 26 mM $NaHCO_3$, 2 m$MCaCl_2$, 10 mM dextrose, pH 7.5) before the experiment.

Optic nerve electrophysiology*:* Compound action potentials (CAP) from dysmyelinated nerves (Shiverer mice, $Mbp^{shi/shi}$) and myelinated nerves (litermate controls, $Mbp^{+/shi}$ and $Mbp^{+/+}$) were recorded using suction electrodes as described by Stys *et al* (Stys et al., 1991). Briefly, the dissected optic nerve was placed inside a chamber containing oxygenated (5 % CO_2, 95 % O_2) aCSF (300 µL, 126 mM NaCl, 3 mM KCl, 2 mM $MgSO_4$, 26 mM $NaHCO_3$, $CaCl_2$, D-glucose 10 mM) between two suction electrodes (stimulus and recording electrodes) forming a tight seal on each end. Two additional electrodes were placed in the bath for reference. A supramaximal pulse (250 mV, 20 µs) was applied at the stimulating end of the nerve. The resulting CAP was amplified from the recording electrode using a high impedance low-noise amplifier (EG&G Princeton Applied Research Corporation) sampled at 100 kHz and filtered at 10 kHz. To assess the effects of the 4-AP derivatives on the CAP, the drug under study was added in incremental concentrations to the recording chamber after the CAP was allowed to stabilize for 5 min while

pulsing repeatedly. After each measurement, the chamber was washed for 5 min (flow 1 mL/min) with oxygenated aCSF. The study was conducted at 22.2 ± 1.3 °C to allow for slower conduction and the temperature was monitored throughout the experiment. CAP recordings were acquired with a SBC6711 board (Innovative Integration) controlled by in-house written software. Analysis of the traces was done using an in-house software. The half-maximal effective concentration (EC_{50}) for each drug was calculated by plotting the final over initial amplitude *vs.* concentration and fitted to the Hill equation using Origin.

Supplemental Methods References

Hoshi, T., Zagotta, W.N., and Aldrich, R.W. (1990). Biophysical and molecular mechanisms of Shaker potassium channel inactivation. Science *250*, 533-538.

Mochizuki, A., Nagata, T., Kanno, H., Suzuki, M., and Ohta, T. (2011). 2-aminomethylphenylamine as a novel scaffold for factor Xa inhibitor. Bioorganic & medicinal chemistry *19*, 1623-1642.

Spivey, A.C., Fekner, T., Spey, S.E., and Adams, H. (1999). Configurationally Stable Biaryl Analogues of 4-(Dimethylamino)pyridine: A Novel Class of Chiral Nucleophilic Catalysts. The Journal of Organic Chemistry *64*, 9430-9443.

Stefani, E., and Bezanilla, F. (1998). Cut-open oocyte voltage-clamp technique. Methods Enzymol *293*, 300-318.

Stys, P.K., Ransom, B.R., and Waxman, S.G. (1991). Compound action potential of nerve recorded by suction electrode: a theoretical and experimental analysis. Brain research *546*, 18-32.

Wang, Y., Wu, C., Caprariello, A.V., Somoza, E., Zhu, W., Wang, C., and Miller, R.H. (2009). In vivo quantification of myelin changes in the vertebrate nervous system. The Journal of neuroscience : the official journal of the Society for Neuroscience *29*, 14663-14669.

Sup. Fig. 1. [^{14}C]4-AP autoradiography of control and demyelinated spinal cords. Increased uptake of [^{14}C]4-AP in the spinal cord of DTA mice. DTA mice show prominent demyelination in the spinal cord 7 weeks post injection of tamoxifen. *Left:* LFB staining showing normal myelin in control sections and severe demyelination in DTA sections. *Right:* [^{14}C]4-AP autoradiography showing high signal in the dorsal and ventral columns of DTA sections respect to control (n = 2 animals, 1 control and 1 demyelinated; 2 sections per animal).

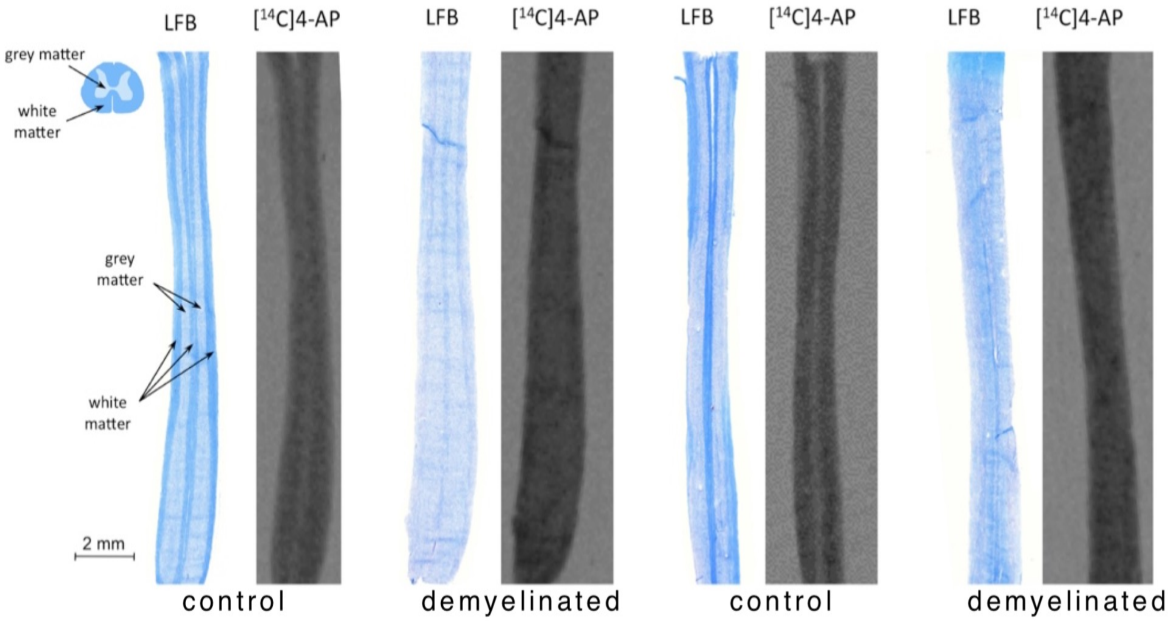

Sup. Fig. 2. **Serial autoradiography and LFB images of the cerebellum of a demyelinated rat**. LPC was injected on the right side of the cerebellum. Sectioned area spans from bregma -13.1 mm to -9.1 mm (caudal to rostral). Each section is 60 µm thick and 120 µm apart. The demyelinated lesion is present from section #16 to #28. There is very high correlation between the autoradiography where the lesion appears as a focal increase in signal and the LFB staining where the lesion appears devoid of LFB stain. Zoom square size corresponds to 7.2 x 7.2 mm.

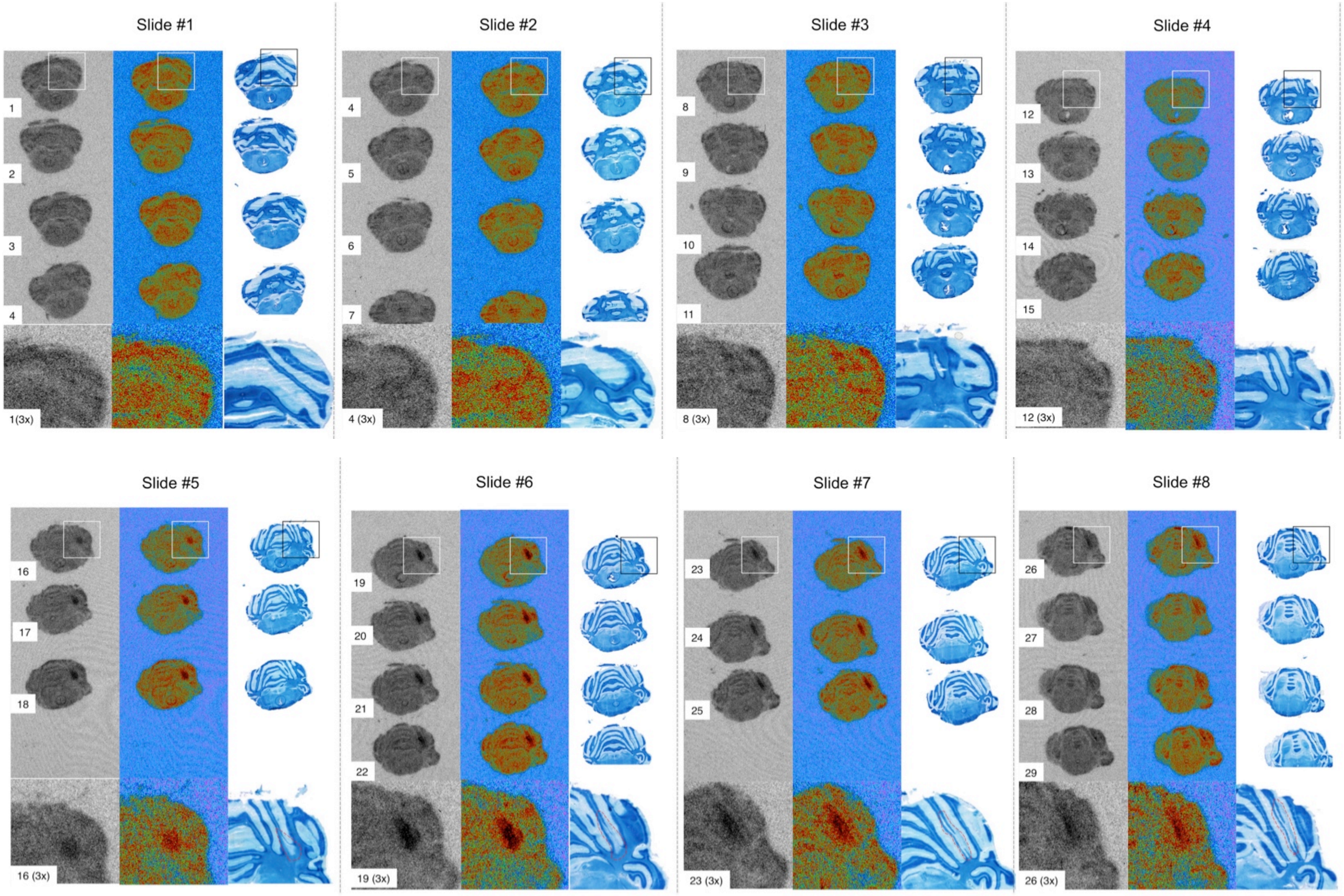

Sup. Fig. 3. Time series images and time-activity curves of kidney and bladder.

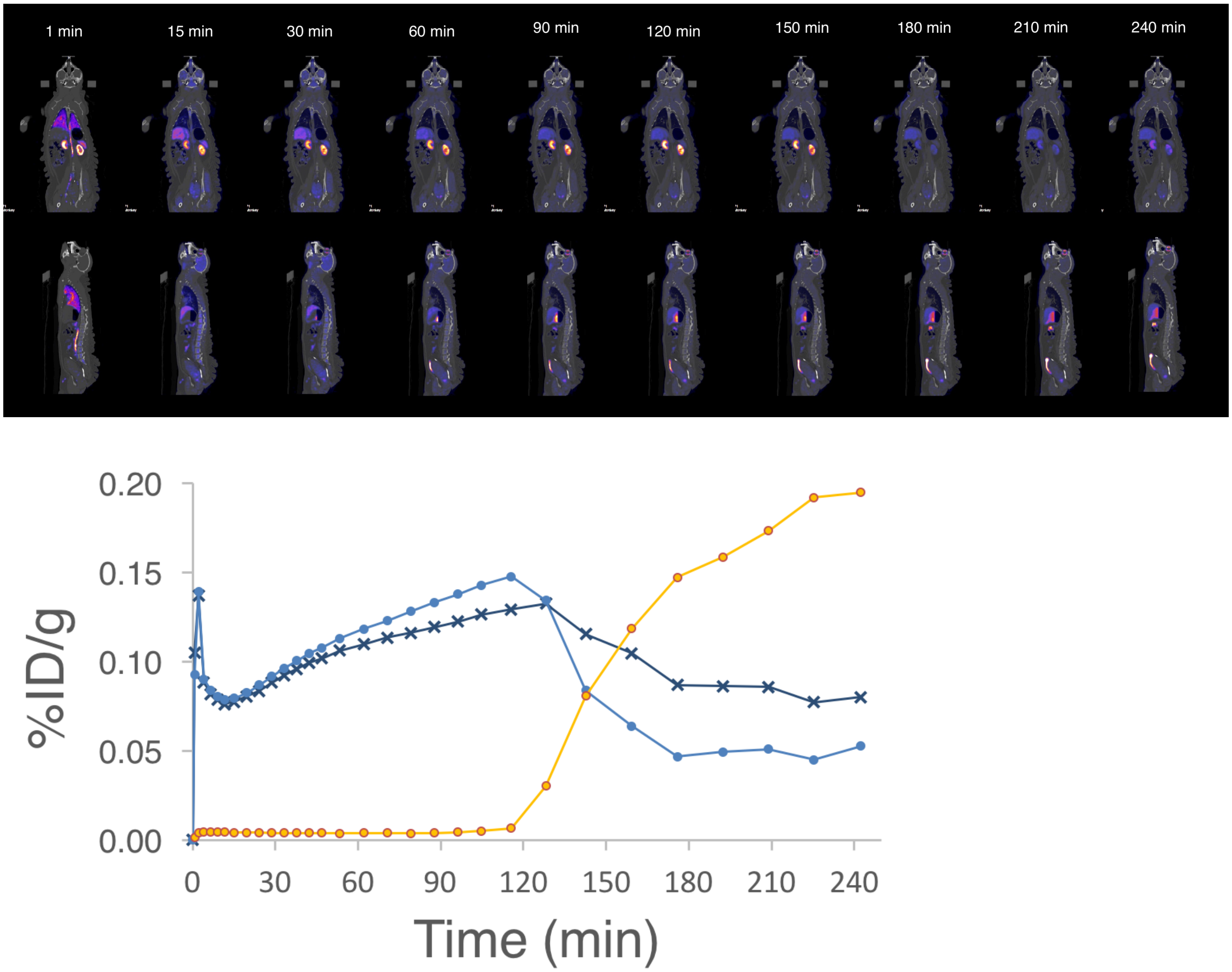

Sup. Fig. 3. Eye uptake in pigmented and albino rats. PET/CT images of pigmented (Long Evans) and albino (Sprague Dawley) rats. There is high eye signal on the pigmented rat but not on the albino rat.

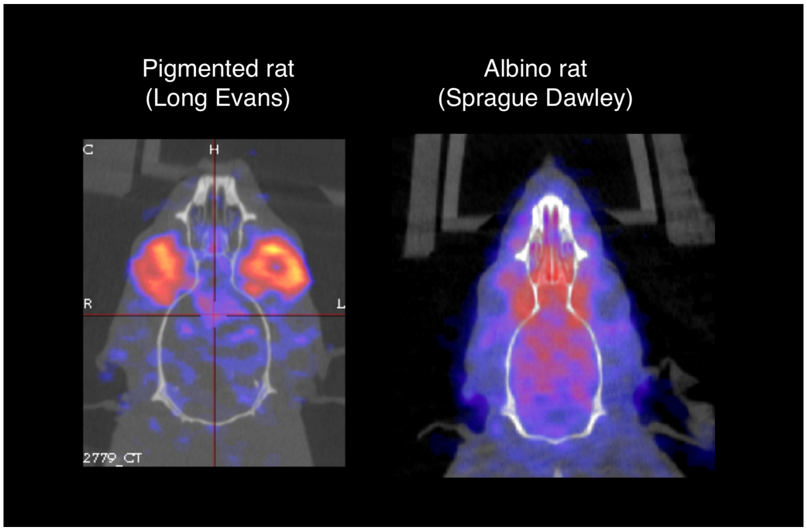

Sup. Fig. 4. PET images of monkey eyes (22 - 54 min). Radioactivity accumulates in the iris and retinal pigmented epithelium (RPE). Areas with highest concentration of melanin. Monkeys have approximately 10 times more melanin in those areas than humans (65).

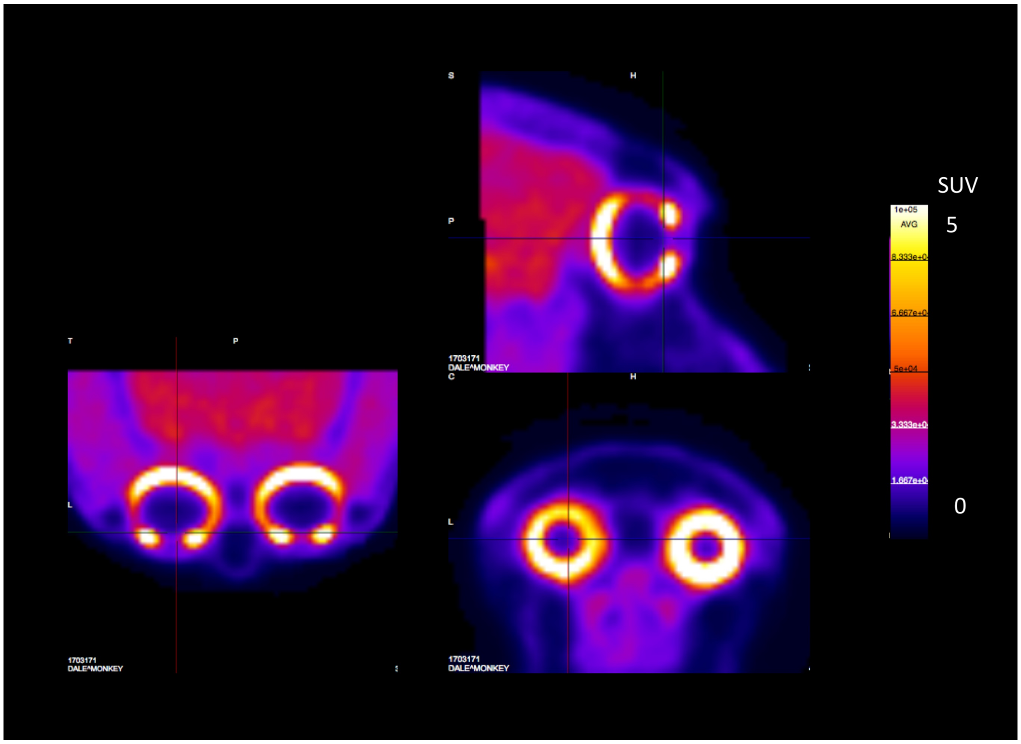

Sup. Fig. 6. Time dependent PET images of the Rhesus brain

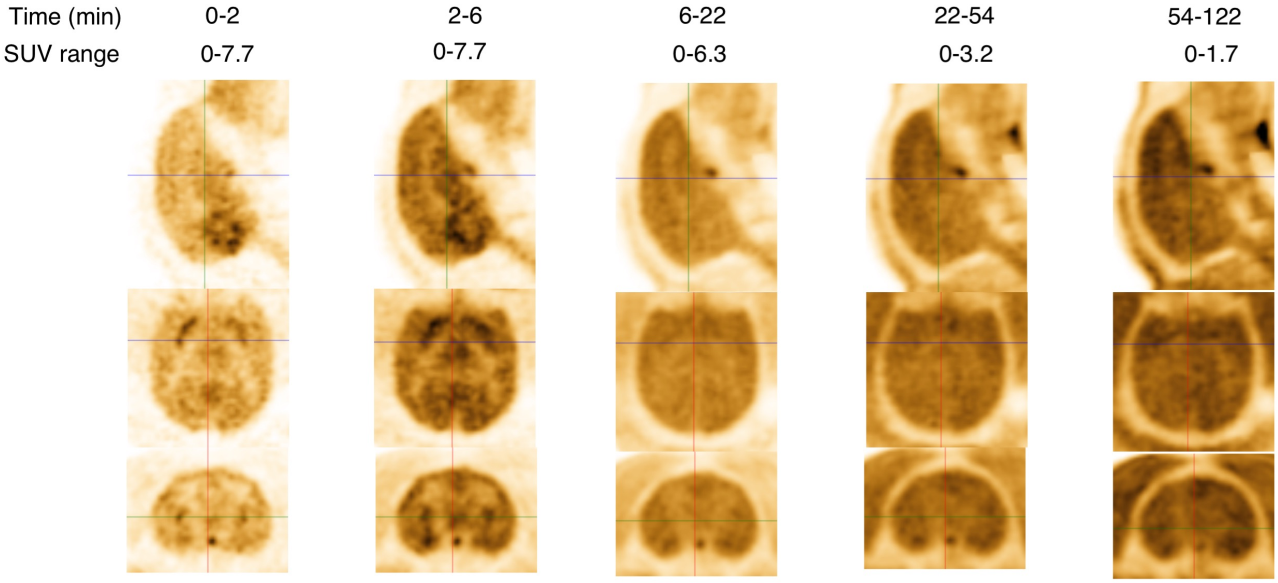

Sup. Fig. 7. Brain TACs after preinjection of 0.1 mg/kg of [^{18}F]3-F-4-AP

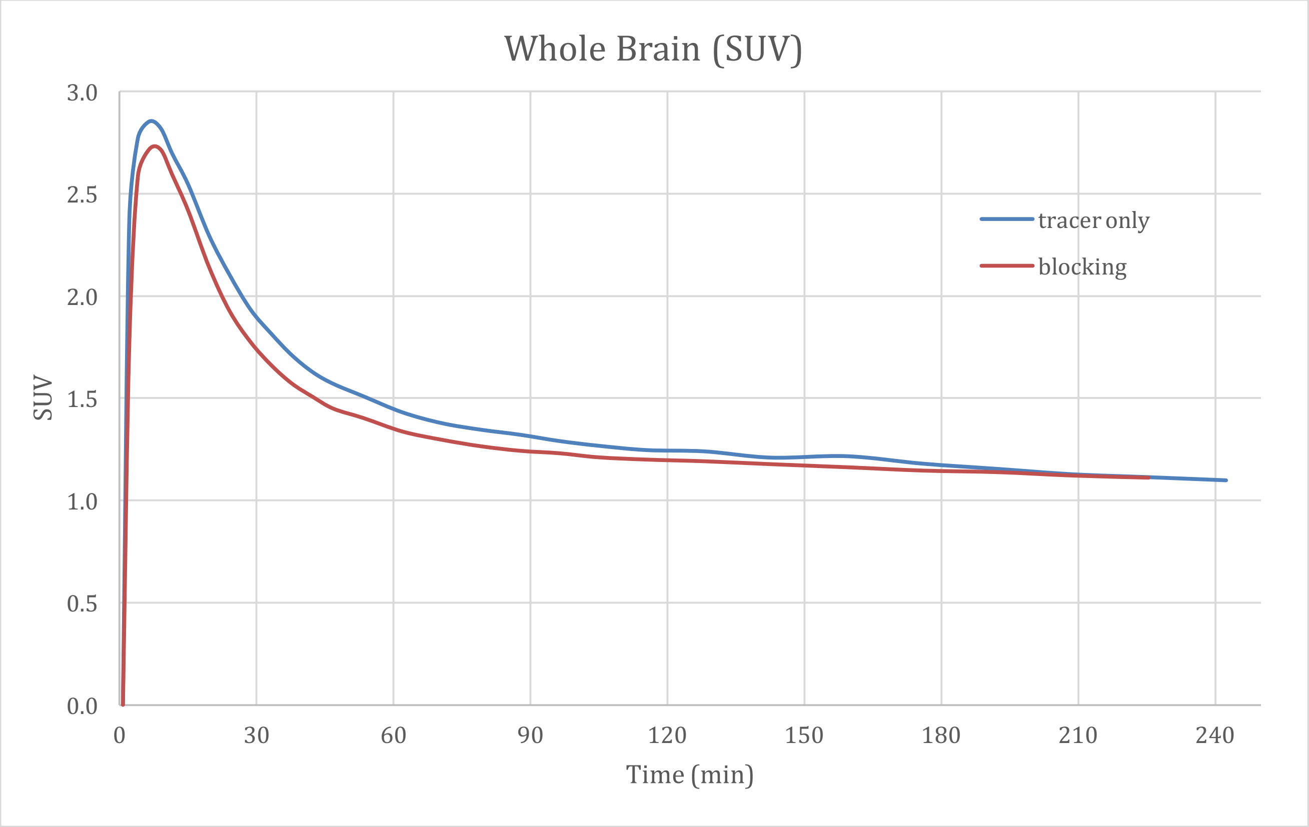

SUPPLEMENTAL MOVIES

Sup. movie 1. Rotating maximum intensity projection of a whole-body monkey scan (2-6 min post injection).

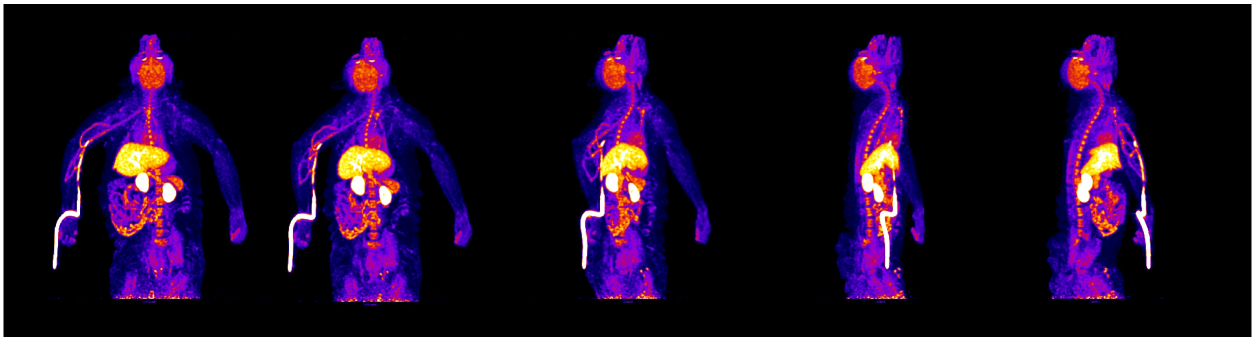

Still frames, please see supplemental file for movie.

Sup. movie 2. Dynamic PET images time series. Each frame represents one cycle through 3 bed positions (2 × 15 secs, 4 × 30 secs, 8 × 60 secs, 8 × 120 secs, 8 × 240 secs).

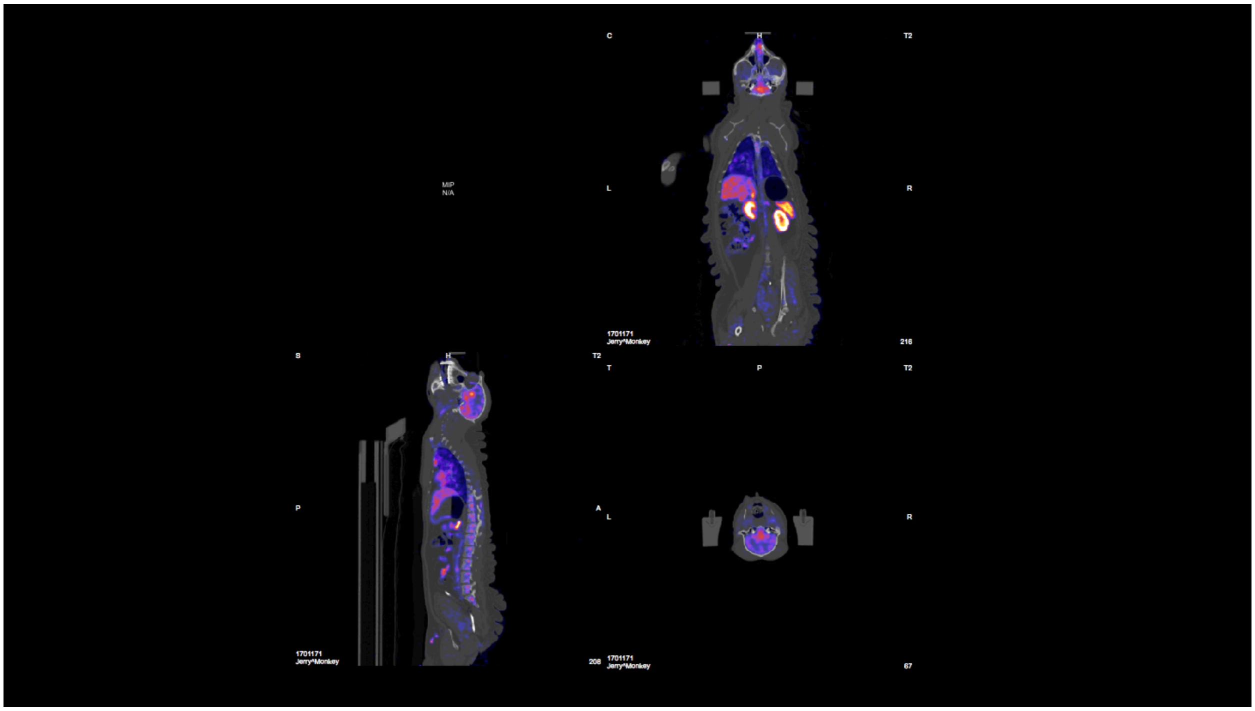

Still frame, please see supplemental file for movie.

Sup. movie 3. Rotating view of the monkey eyes and forehead.

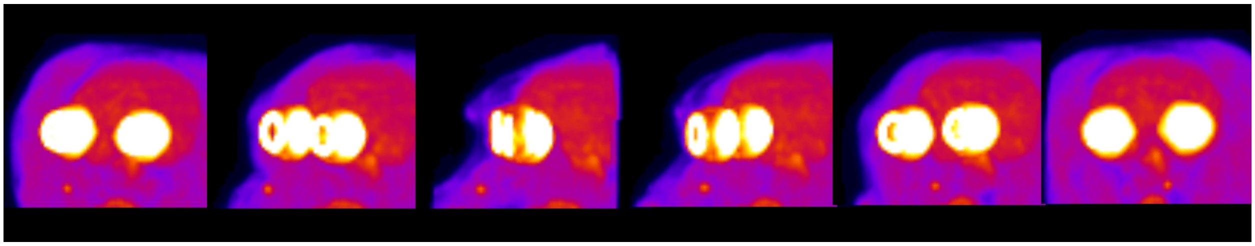

Still frames, please see supplemental file for movie.

SPECTRAL DATA:

tert-butyl N-[3-(fluoromethyl)pyridin-4-yl]carbamate **(9)**

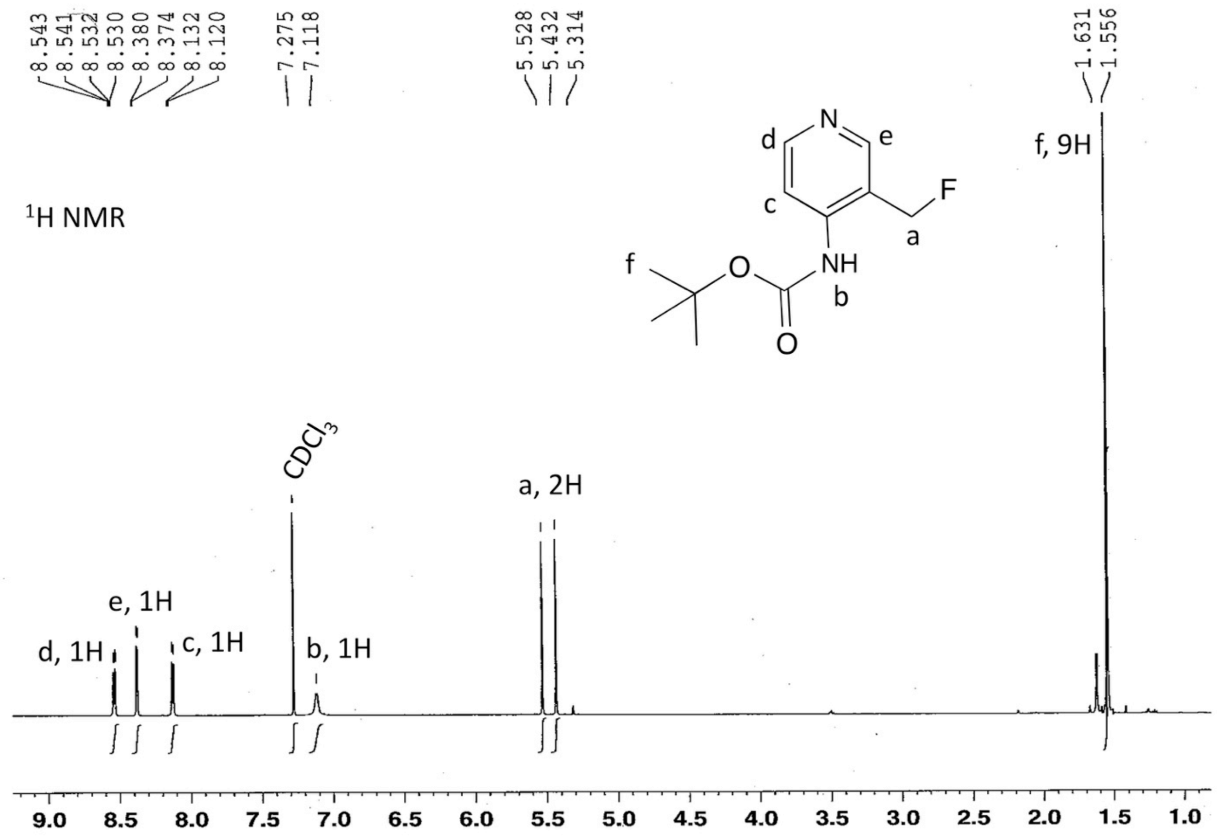

tert-butyl N-[3-(fluoromethyl)pyridin-4-yl]carbamate **(9)**

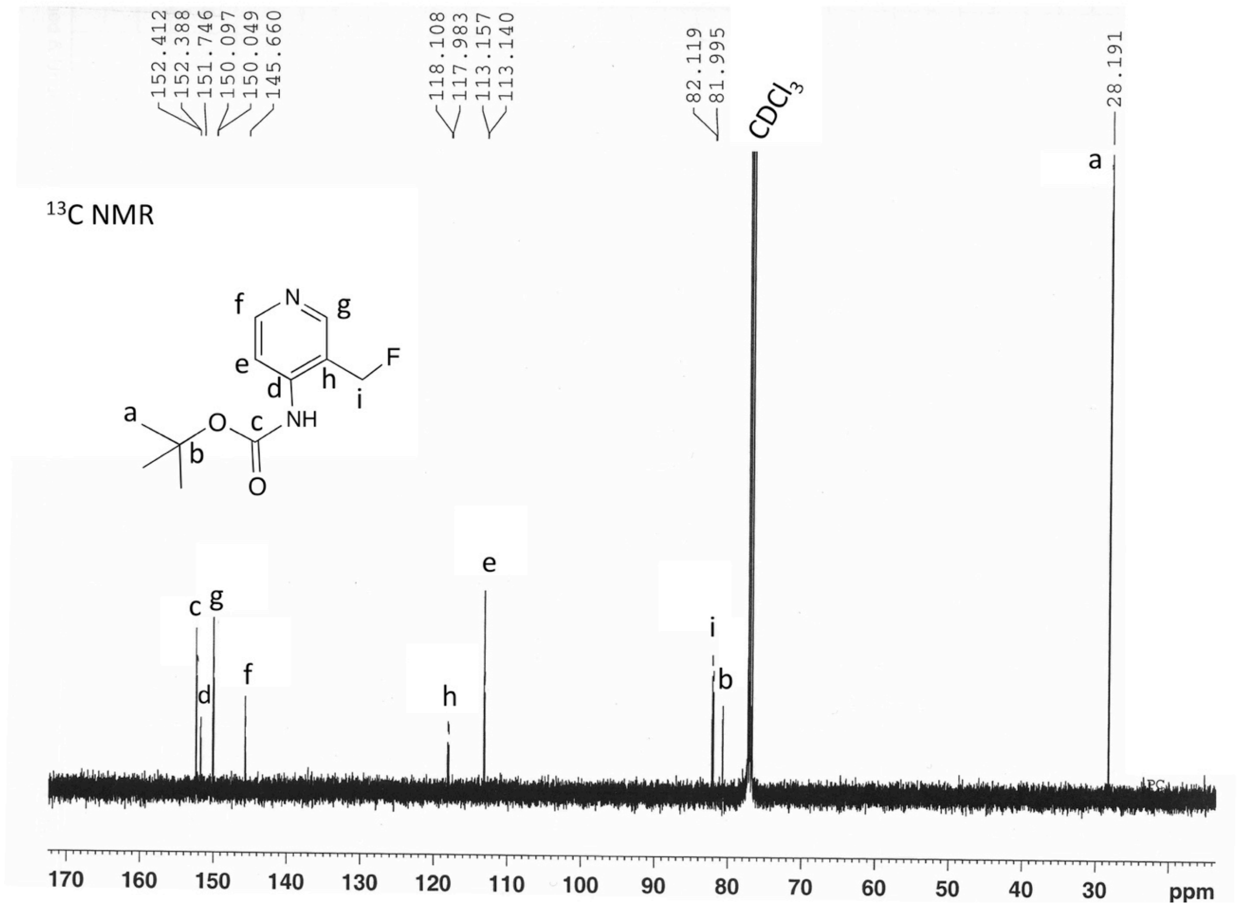

tert-butyl N-[3-(fluoromethyl)pyridin-4-yl]carbamate **(9)**

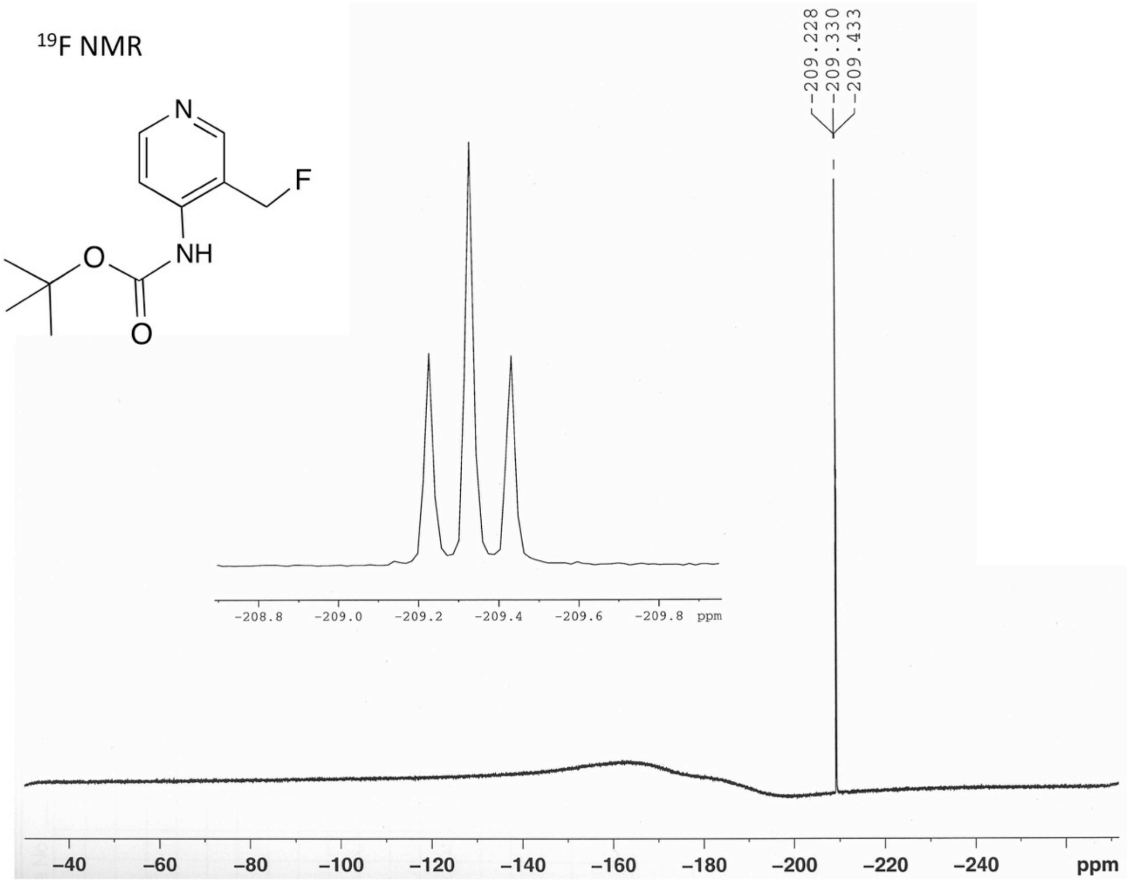

tert-butyl N-[3-(fluoromethyl)pyridin-4-yl]carbamate **(9)**

HRMS

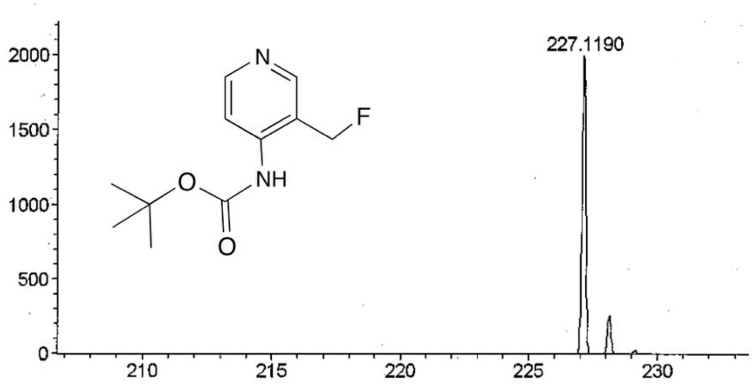

Chemical Formula: $C_{11}H_{15}FN_2O_2$ Exact Mass: 226.1118

3-fluoromethyl-4-aminopyridine (**5**)

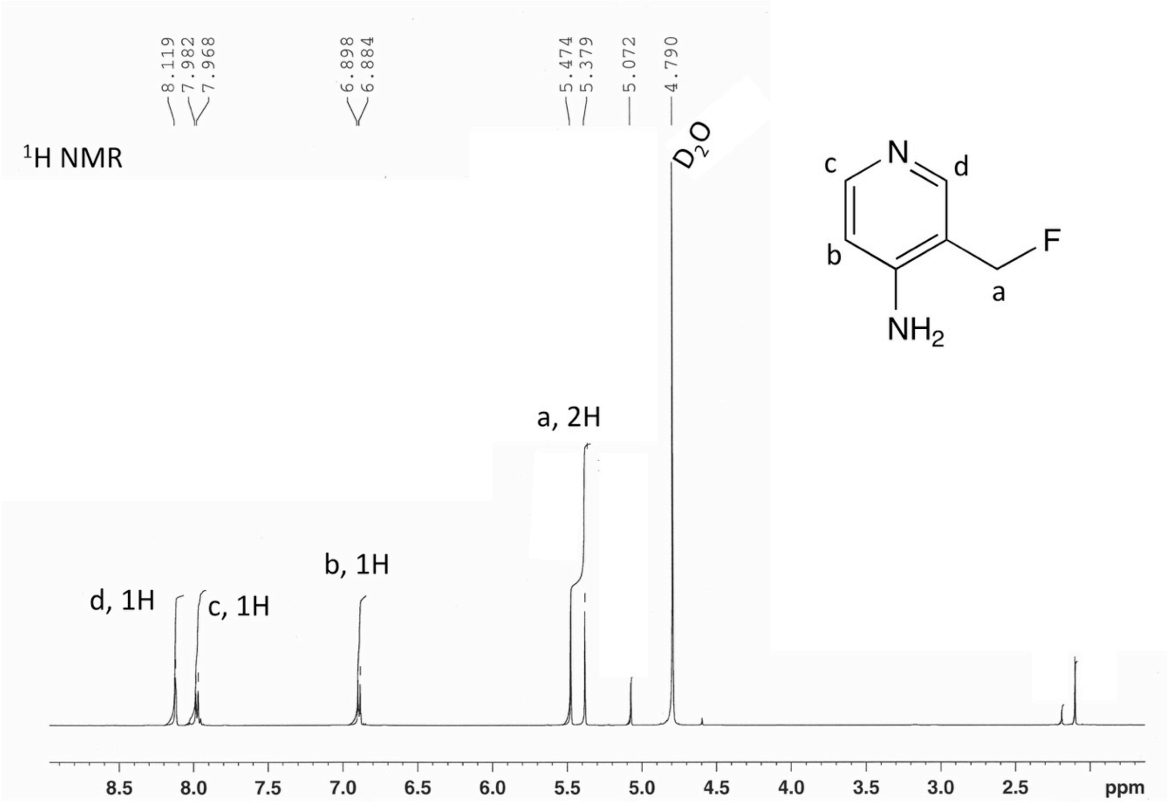

3-fluoromethyl-4-aminopyridine (**5**)

^{19}F NMR

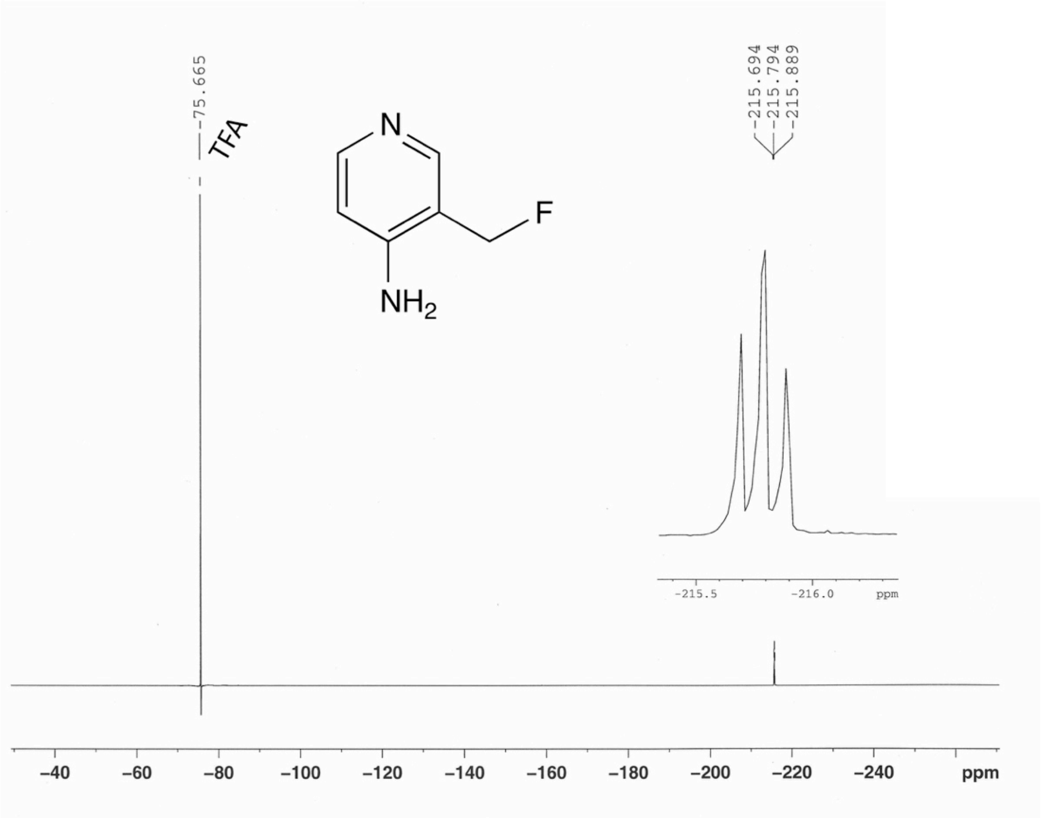

3-fluoromethyl-4-aminopyridine (**5**)

HRMS

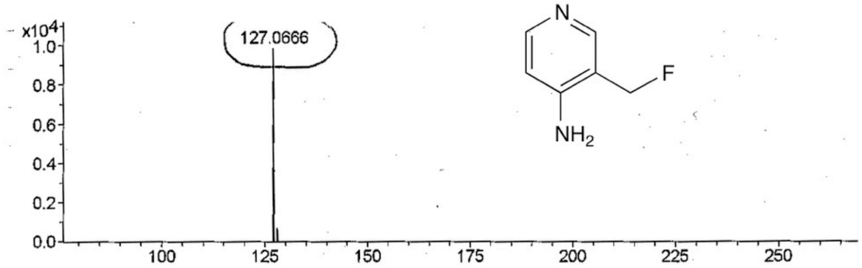

Meas. m/z	#	Formula	m/z	err [ppm]	Mean err [ppm]	rdb	N-Rule	e⁻ Conf
127.0679	1	C 6 H 8 F N 2	127.0666	-10.3	-10.1	3.5	ok	even

tert-butyl N-[3-(2-hydroxyethyl)pyridin-4-yl]carbamate (**11**)

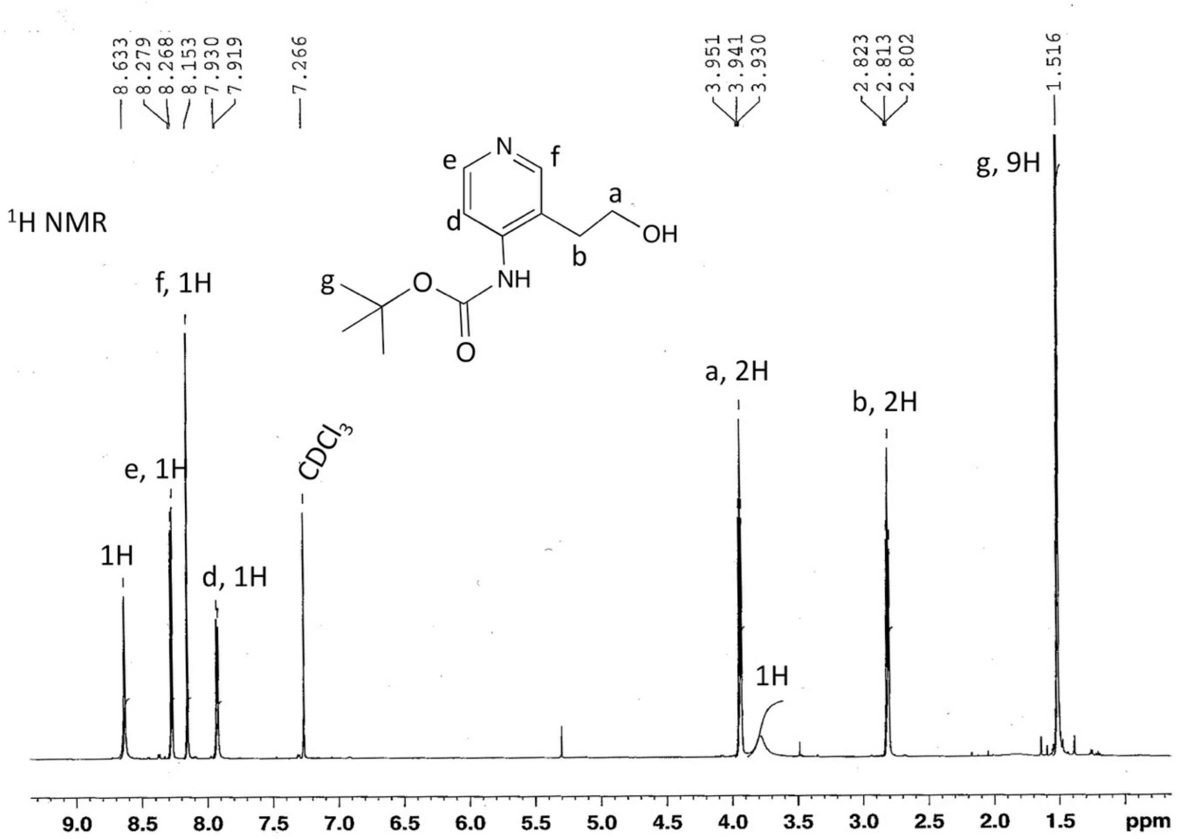

tert-butyl N-[3-(2-fluoroethyl)pyridin-4-yl]carbamate (**12**)

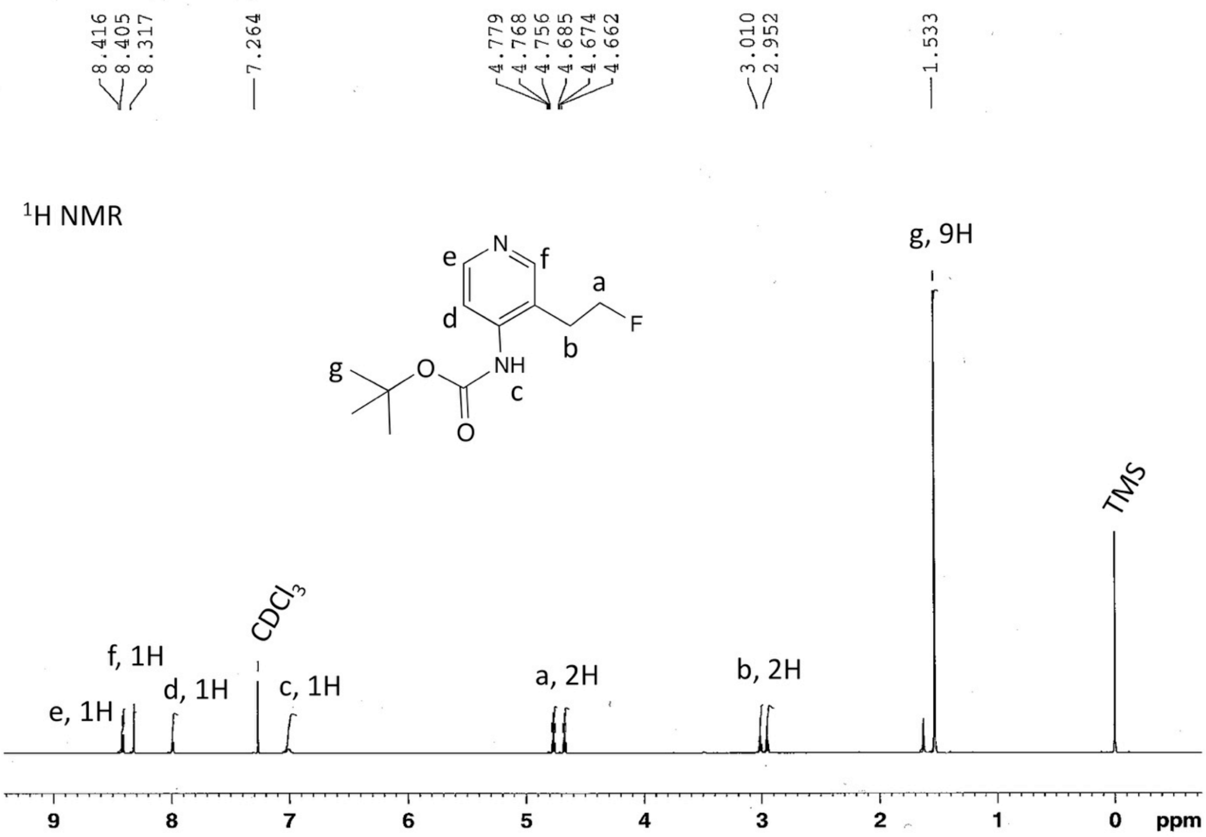

tert-butyl N-[3-(2-fluoroethyl)pyridin-4-yl]carbamate (**12**)

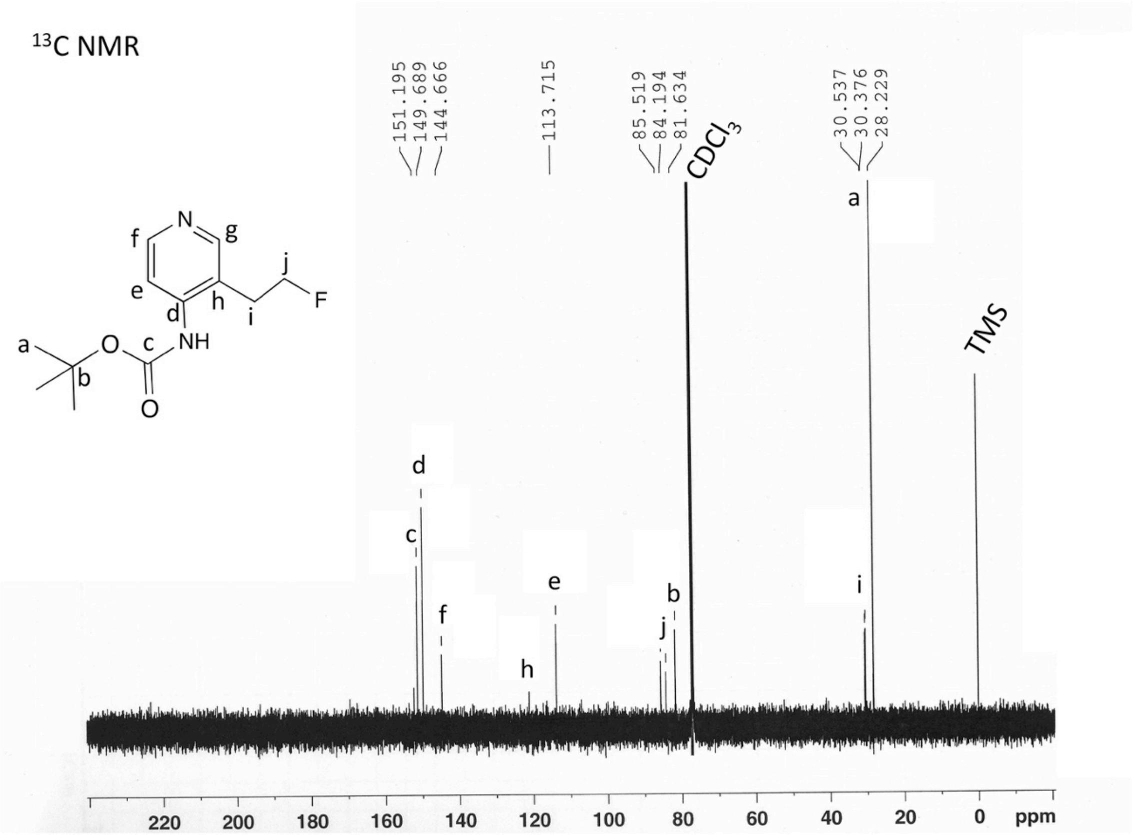

tert-butyl N-[3-(2-fluoroethyl)pyridin-4-yl]carbamate (**12**)

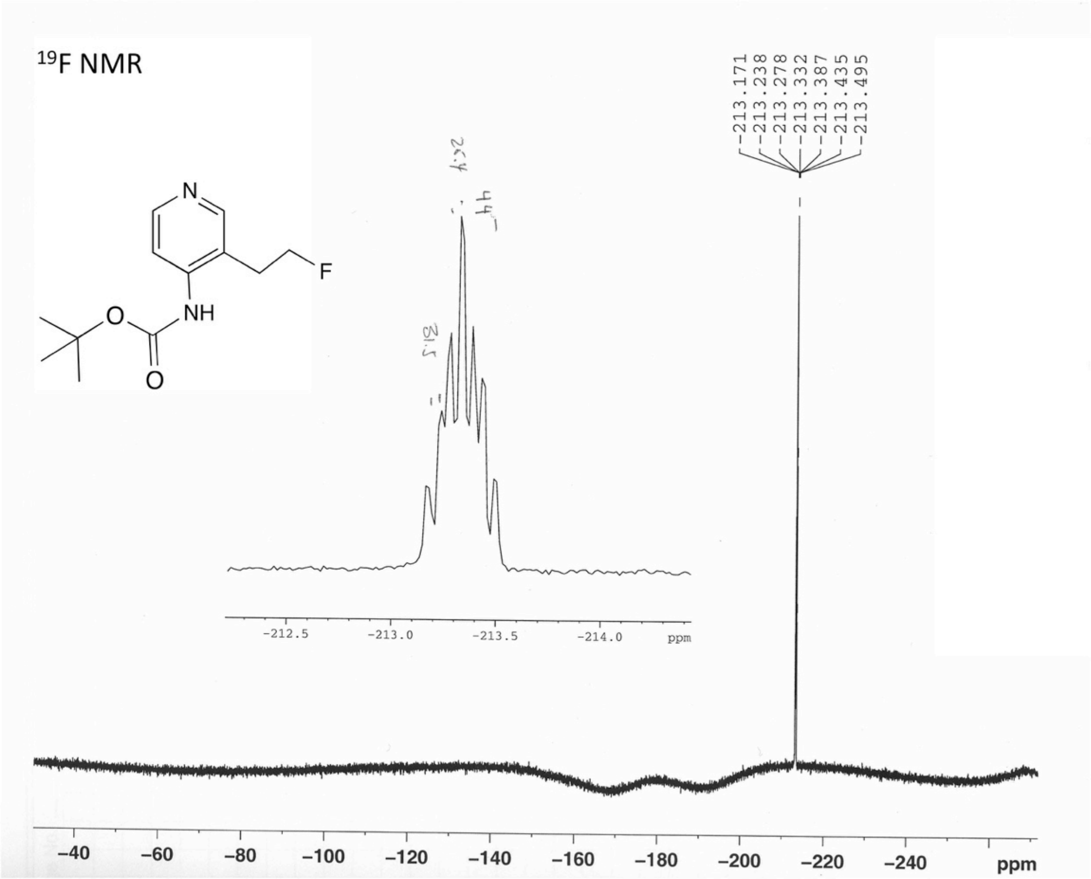

tert-butyl N-[3-(2-fluoroethyl)pyridin-4-yl]carbamate (**12**)

HRMS

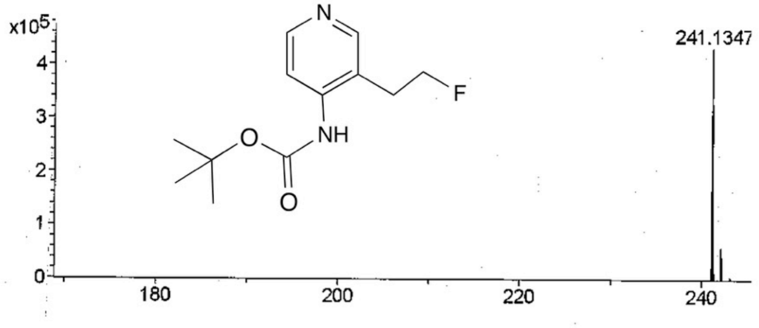

Meas. m/z	#	Formula	m/z	err [ppm]	Mean err [ppm]
241.1354	1	C 12 H 18 F N 2 O 2	241.1347	-3.1	-3.2

3-fluoroethyl-4-aminopyridine (**6**)

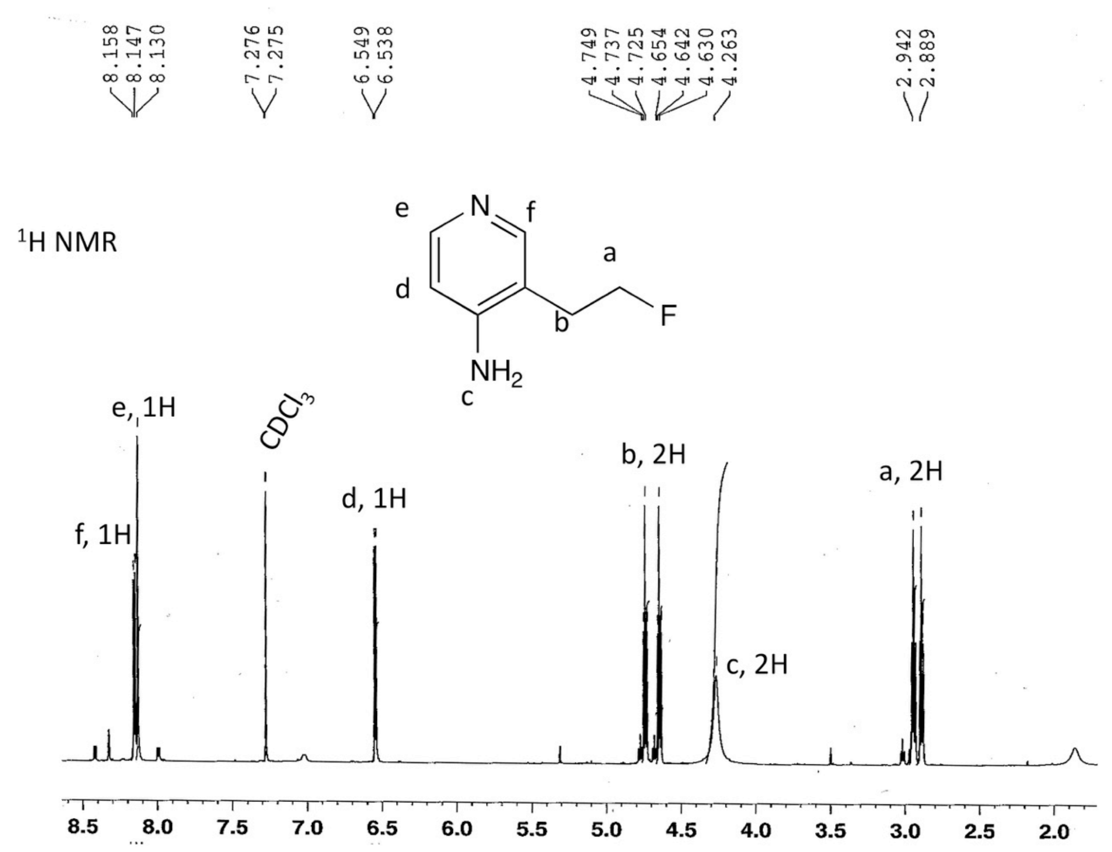

3-fluoroethyl-4-aminopyridine (**6**)

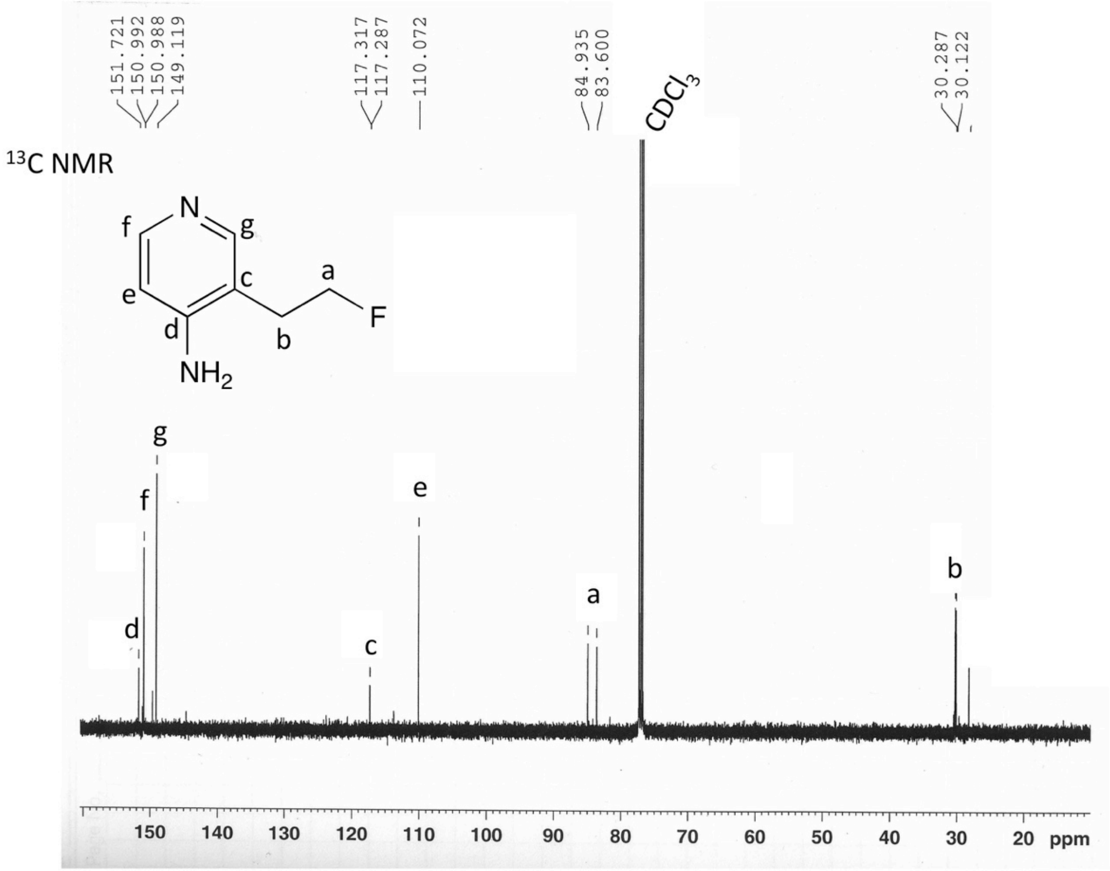

3-fluoroethyl-4-aminopyridine (**6**)

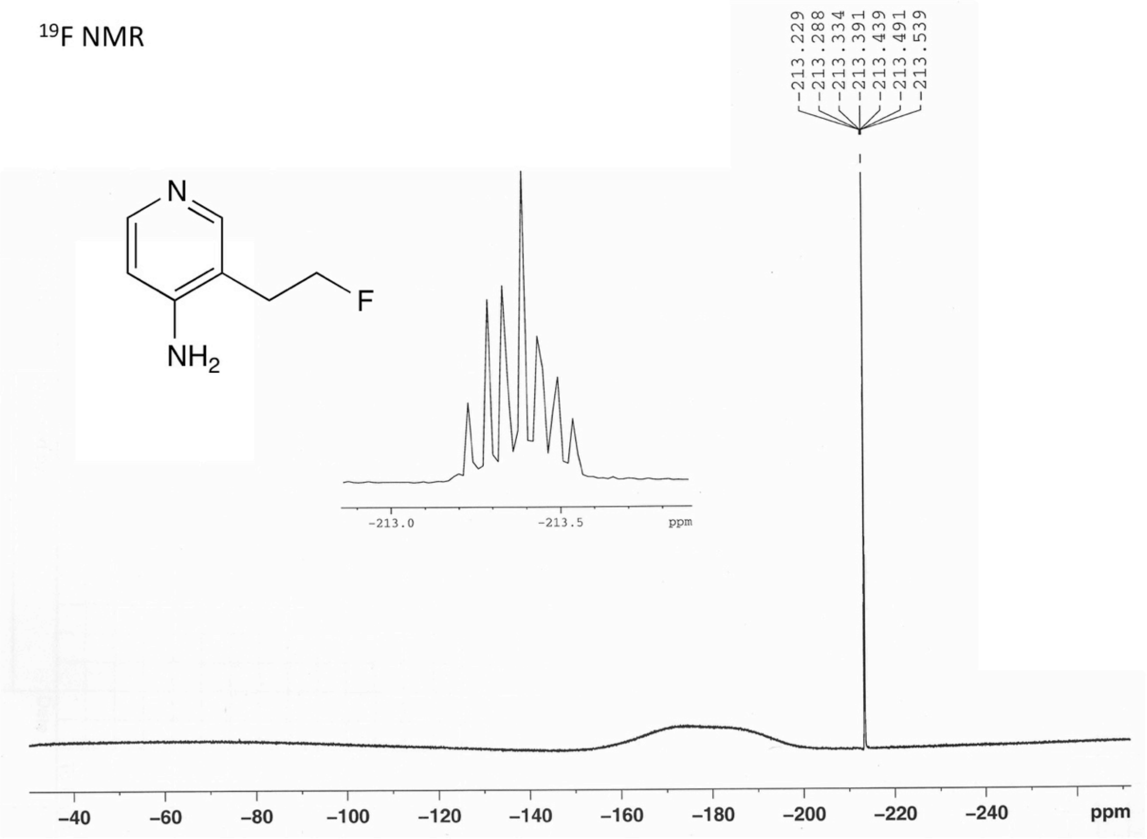

3-fluoroethyl-4-aminopyridine (**6**)

HRMS

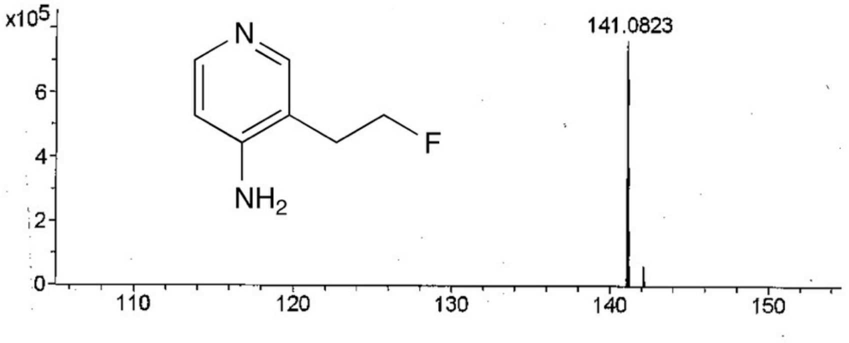

/leas. m/z	#	Formula	m/z	err [ppm]	Mean err [ppm]	rdb
141.0831	1	C 7 H 10 F N 2	141.0823	-6.3	-6.3	3.5
